# Epidemiology of Mental Health Attendances at Emergency Departments: Systematic Review and Meta-Analysis

**DOI:** 10.1371/journal.pone.0154449

**Published:** 2016-04-27

**Authors:** Helen Barratt, Antonio Rojas-García, Katherine Clarke, Anna Moore, Craig Whittington, Sarah Stockton, James Thomas, Stephen Pilling, Rosalind Raine

**Affiliations:** 1 NIHR CLAHRC North Thames, Department of Applied Health Research, University College London, London, United Kingdom; 2 Centre for Outcomes Research and Effectiveness (CORE), Research Department of Clinical, Educational and Health Psychology, University College London, London, United Kingdom; 3 Division of Psychiatry, University College London, London, United Kingdom; 4 Department of Psychiatry, University of Oxford, Oxford, United Kingdom; 5 Institute of Education EPPI-Centre, London, United Kingdom; Yokohama City University, JAPAN

## Abstract

**Background:**

The characteristics of Emergency Department (ED) attendances due to mental or behavioural health disorders need to be described to enable appropriate development of services. We aimed to describe the epidemiology of mental health-related ED attendances within health care systems free at the point of access, including clinical reason for presentation, previous service use, and patient sociodemographic characteristics.

**Method:**

Systematic review and meta-analysis of observational studies describing ED attendances by patients with common mental health conditions.

**Findings:**

18 studies from seven countries met eligibility criteria. Patients attending due to mental or behavioural health disorders accounted for 4% of ED attendances; a third were due to self-harm or suicidal ideation. 58.1% of attendees had a history of psychiatric illness and up to 58% were admitted. The majority of studies were single site and of low quality so results must be interpreted cautiously.

**Conclusions:**

Prevalence studies of mental health-related ED attendances are required to enable the development of services to meet specific needs.

## Introduction

In recent years, a number of initiatives have been developed to improve outcomes and experience for patients who attend hospital Emergency Departments (EDs) due to mental or behavioural health disorders. This is partly because of concerns about the quality of care for this patient group,[1] but also the need to ensure parity of response to mental and physical health emergencies.[2] In England, for example, the National Health Service (NHS) Mandate for 2014/15 stated that services for patients in mental health crisis should be as accessible, responsive and high quality as emergency services for other patients.[3] Yet a recent report by the Care Quality Commission demonstrated that there are clear variations in the help, care and support available to people in crisis and many patients still have a poor experience of care because services fail to meet their needs.[1] The first step towards improving health services requires high quality information about clinical need to ensure that services are designed to meet patients’ needs. However, recent commissioning guidance in the UK[4]^,^[5] was based on a sample of Medicare patients in the United States who attended hospitals in 1999.[6] This study also looked only at the prevalence of depressive symptoms. To ensure generalisability, we needed to consider whether the use of these data was valid, given the differences between the US health care system and systems such as the NHS, where care is free to all at the point of use, independent of ability to pay. In order to inform service change and the development of new models of care, we undertook a systematic review and meta-analysis of the epidemiology of mental health-related Emergency Department (ED) attendances by adults within publically supported health care systems such as the English NHS and similar. We aimed to quantify the proportion of ED attendances that are due to mental or behavioural disorders and to identify patient clinical and sociodemographic characteristics associated with this type of attendance.

## Materials and Methods

This analysis formed part of a series of linked reviews examining the epidemiology of mental or behavioural health disorders in EDs. Here, we present a systematic review of observational studies describing the overall population of patients attending hospital Emergency Departments because of a mental or behavioral health issue. Studies describing the epidemiology of specific mental health conditions will be examined elsewhere.

### Search strategy and selection criteria

Electronic database searches were conducted in Embase, Medline, PreMedline, PsycINFO and CINAHL with an English language restriction (see S1 Appendix for the search strategy containing a full list of the search terms used). The first National Service Framework for mental health was published in 1999, redefining care standards across the UK. We therefore limited the search to studies published since 1 January 2000.

We searched for studies describing patients who attended a hospital ED with a primary diagnosis of either one of more mental and behavioural disorders (F01-F79 of the International Classification of Diseases, 10^th^ edition) or self-harm (X60-X84). Studies also had to report one or more epidemiological measure, for example the frequency, incidence, occurrence, or prevalence of mental health-related attendances to the ED. As this study relates to adult ED attendances, we did not include ‘disorders of psychological development’ (F80-89) or ‘behavioural and emotional disorders with onset usually occurring in childhood and adolescence’ (F90-F98) in the search. All records identified from the searches were uploaded to EPPI-Reviewer 4[7] for screening.

Inclusion criteria applied at the screening stage stated that studies of mental health-related attendances must:

1. Describe services in the UK, the rest of Western Europe, Canada, or Australasia (as these were considered most comparable to the English NHS, where care is free at the point of use);
2. Describe a cohort, case-control, cross-sectional or ecological study; and
3. Relate to patients aged 18 or over.

We employed a text mining and machine learning method, known as ‘active learning’,[8] using the systematic review software EPPI-Reviewer 4 to screen titles and abstracts.[7] The primary goal of text mining was to retrieve information from unstructured text and to present the distilled knowledge to users in a concise form.[9] The machine learned iteratively—from human interaction—to distinguish between relevant, and irrelevant citations during the screening phase of the systematic review. It did this by ranking citations in order of relevance, and presenting them to the reviewer for manual screening. After a small number had been manually screened (e.g. 25 citations), the machine re-ordered the list, taking into account everything that had been screened thus far. Thus, rather than screening the documents in no particular order, those most similar to the studies already selected were moved to the top of the list, increasing the probability that the next document viewed would be selected for further review. We truncated the screening process at the point when 1000 titles and abstracts were consecutively excluded, and therefore the rate of inclusion had dropped to less than 0·1%.[10]

The full-texts of remaining records were then screened by HB, with any queries about inclusion resolved through discussion with a second reviewer (ARG). Duplicates of articles were removed, and studies including the same patients were linked.

### Quality assessment

There is no consensus about the use of rating methods for the quality assessment of epidemiological studies, particularly those reporting cross-sectional observational data.[11] We therefore developed a quality assessment measure for the purposes of this review, which drew on the Newcastle-Ottawa Scale for assessing the quality of non-randomised studies;[12] the STROBE checklist for the reporting of cohort, case-control, and cross-sectional studies;[13] and an additional check-list specifically for the appraisal of cross-sectional studies.[14] Included studies were each rated as good, fair or poor in ten key domains of quality: clarity of focus; appropriateness of method; definition of study population; measures to reduce bias; data collection methods; number of study participants; quality assurance measures; data analyses; completeness of discussion; and generalisability of findings. Where insufficient information was available to assess quality in a particular domain, this was noted. We then classified the overall quality of each study as good, fair or poor, taking all ten domains into account. The measure is included in S2 Appendix.

### Data extraction and synthesis of results

We created a spreadsheet in Microsoft Excel to collect relevant epidemiological data from each paper. Two authors extracted data from the first 10% of articles to check the reliability of this tool, and to check agreement between authors (HB and KC). The remaining data extraction, and quality assessment, was performed independently by three authors (HB, KC and ARG). Queries were resolved through discussion and consensus.

Data were summarised both qualitatively and quantitatively. To facilitate this, we extracted data regarding the following characteristics from all included studies: study design (cross-sectional or cohort, and retrospective or prospective); study setting (country; type of emergency department; number of sites; urbanisation); patient selection (target population; sample size; instrument used to code mental health conditions); clinical reason for attendance; past history of mental illness; destination after discharge; patient characteristics (age; gender; and socioeconomic circumstances, for example, measures of deprivation, receipt of benefit payments or health care subsidies, employment, housing status, or education level); and approach to data collection (consecutive attendances; dates of data collection; time span of data collection in days). When possible, data relating to individual patients (who may have attended the ED more than once) were recorded separately from data relating to total numbers of ED attendances. Studies were coded inductively according to their disease focus by one researcher (ARG), who identified natural groupings of papers within the data. Papers were then grouped together according to their primary disease focus to allow analysis by condition. A narrative summary was then created for each of the study characteristics described above.

Where data were available, meta-analyses were conducted to estimate the proportion of mental health-related attendances in relation to the total number of all ED attendances. Data regarding individual patients or total ED attendances were again handled separately. In addition, several meta-analyses were planned to estimate the proportion of attendances due to specific mental or behavioural health disorders (patients or total attendances). The intra-class correlation coefficient and the design effect were estimated. We then used these figures to calculate an effective sample size. This was done to reduce the impact of clustering on the meta-analysis of proportions, assuming that patients within individual studies (for example, patients attending the same hospital) were more similar to each other than they were to those in other studies, attending a different hospital.[15] Proportions were calculated using double arcsine transformations. This was done to create a sampling distribution that was closer to a normal distribution and hence whose sample variance could be better approximated in order to estimate study weights. This approach was chosen because conventional inverse variance methods have been shown to be suboptimal when conducting meta-analyses of small proportions placing undue weight on studies with proportions close to zero and computing negative confidence intervals, for example.[16] Random effects meta-analyses were undertaken in Microsoft Excel using the add-in MetaXL (available at: http://www.epi-gear.com/index_files/metaxl.html). Heterogeneity was estimated using the *I*^*2*^ statistic, where *I*^*2*^ > 50% was considered substantial heterogeneity.[17] Finally, a sensitivity analysis was planned excluding those studies assessed to be of poor overall quality.

## Results

### Description of studies

**Fig 1:**
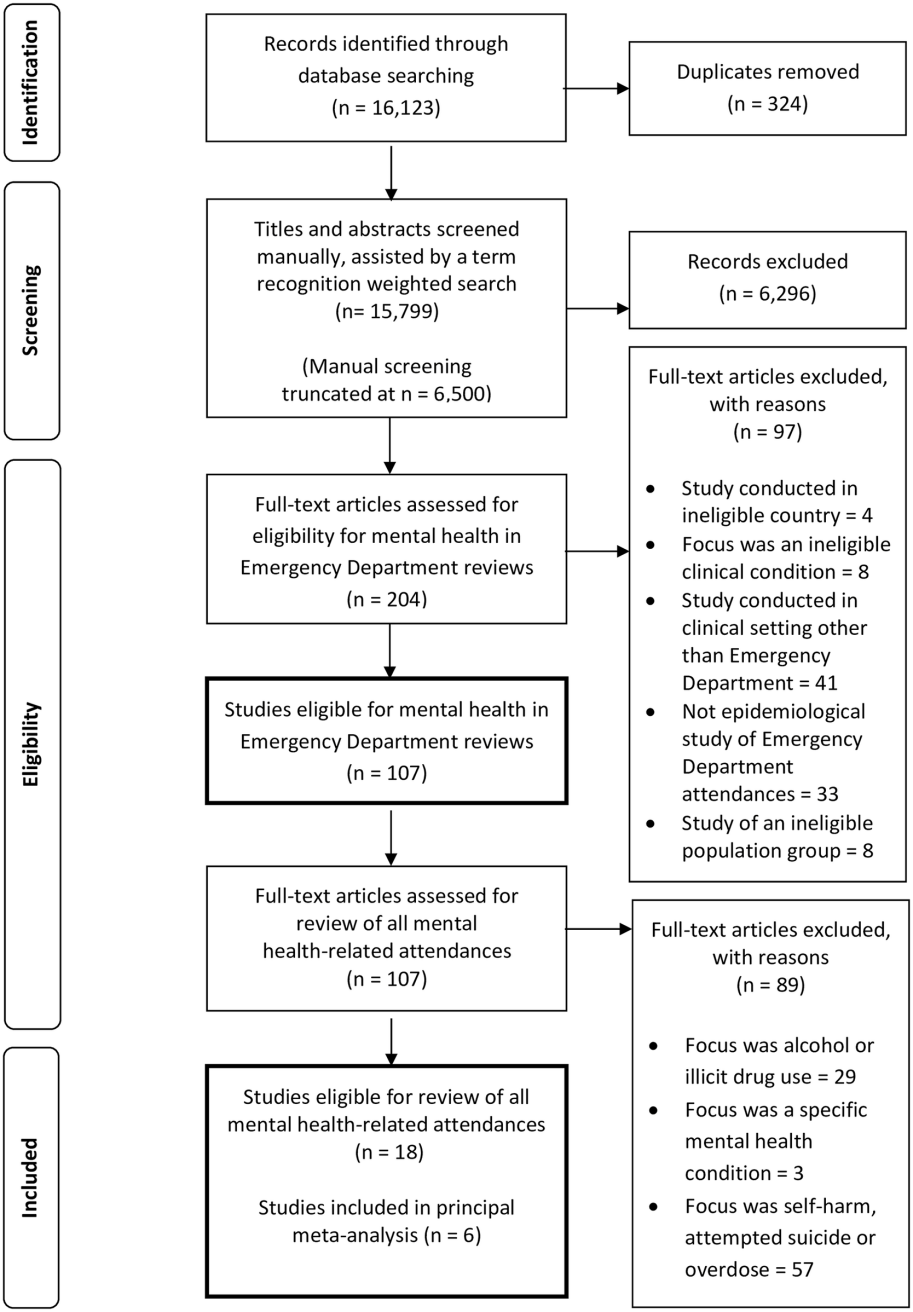
PRISMA Flow Diagram.

The search strategy identified 18 studies that described patients attending hospital Emergency Departments because of mental health conditions, including two conference abstracts (Fig 1).[18,19] Nine studies were conducted in Australia;[20–28] three in Spain;[18,29,30] two in Canada;[31,32] and one in each of the UK,[33] Ireland,[34] Norway[35] and Portugal.[18] Studies took place largely within single emergency departments (n = 14). Five examined attendances to dedicated psychiatric EDs, rather than general departments. Further information about the included studies can be found in the online supplement (see S3 Appendix for an overview and S4 Appendix for characteristics of each study).

The studies differed in the data they reported: whether in terms of total ED attendances, or in terms of individual patients who may potentially have made multiple attendances. Eight reported only attendances; six reported only patients; and four described both types of data. Sample size varied from 168[27] to 290,606[24] ED episodes and 36[19] to 3853[31] individual patients. As detailed in S3 Appendix, studies used a range of instruments to code patient diagnoses, including ICD-9 or 10 (n = 7), DSM-IV (n = 3) and a health professional’s personal assessment (n = 3).

All included studies reported that data were collected on a consecutive sample of eligible patients attending the ED during the study period. Most used a cross-sectional study design (n = 16). In the majority of cases (n = 12), data collection was carried out retrospectively for a specified time period. The length of data collection ranged considerably from 31[19] to 3,652 days.[21]

### Quality assessment

**Fig 2:**
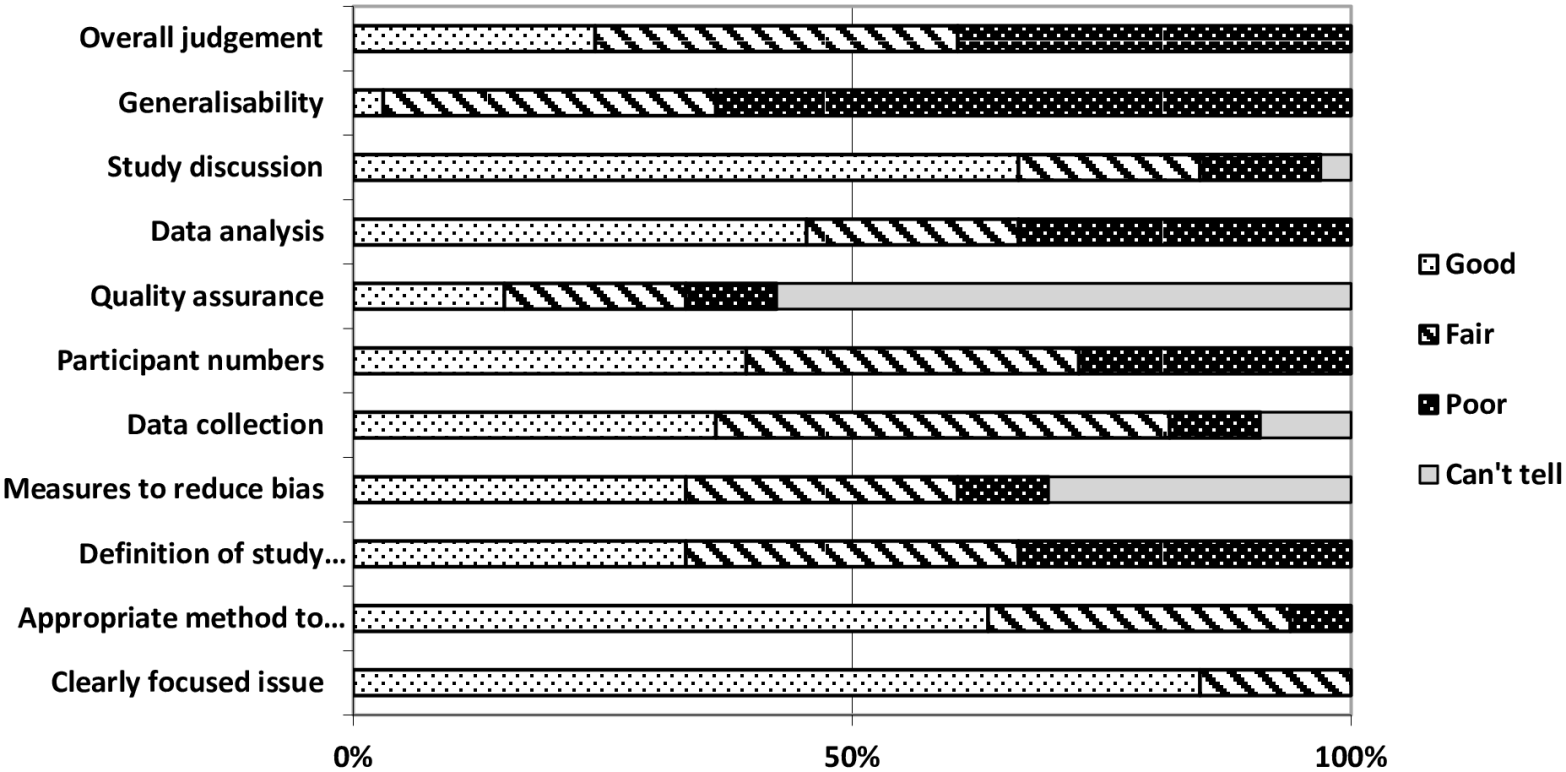
Methodological quality of included studies (n = 18).

Our assessment of the methodological quality of the included studies is summarised in Fig 2. Only three studies were considered to be of good overall quality. Ten were assessed to be of fair quality, whilst the remaining five were poor. Typically more than half of the studies were assessed to be either good or fair with respect to each of the ten individual domains of quality. The generalisability of the findings was assessed as poor in 15/18 studies, usually because the study described a relatively small sample from a single hospital site. Insufficient information was provided in many cases to enable us to assess the quality and robustness of studies in three domains: measures taken to reduce bias (n = 6); data collection processes (n = 5); and quality assurance mechanisms (n = 13). Our ability to assess methodological quality was impacted by a range of factors, for example limited descriptions of how the study population was identified or how analyses were conducted. In addition, many papers provided insufficient information about measures taken by the authors to assure the quality of the data, such as accuracy checking, or how data was actually collected in the ED.

### Proportion of ED attendances related to mental or behavioural health disorders

**Fig 3:**
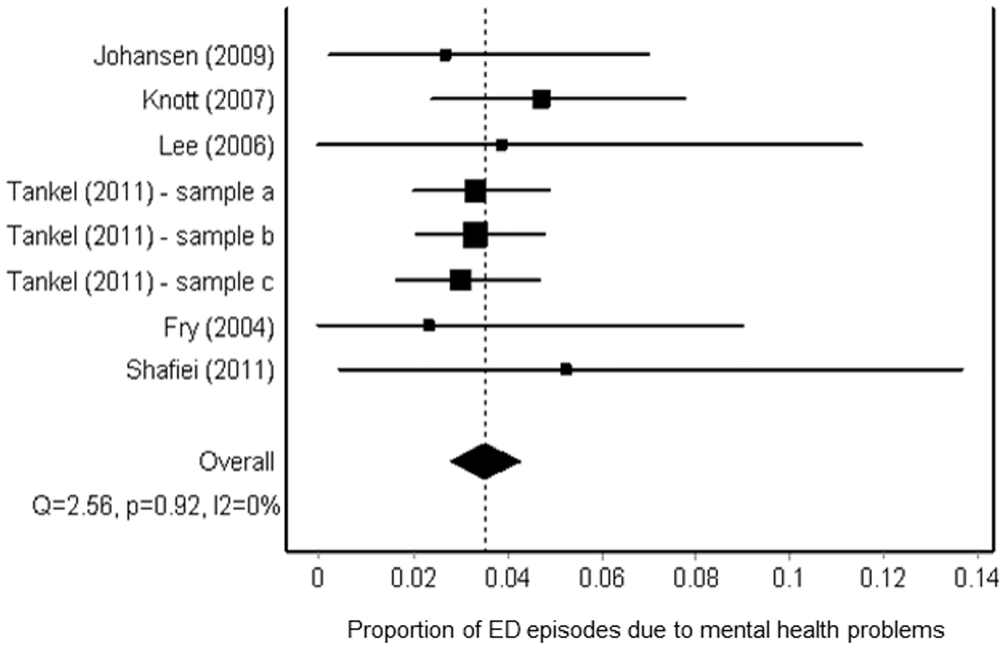
Forest plot (random effects)—proportion of all ED episodes related to mental health disorders. In Fig 3, each of the lines with a square represents one of the studies included in the meta-analysis. Where findings were reported for several different cohorts within one publication, results from each centre/time period/cohort appear separately. The squares are centred on the point estimate of the result from the relevant study. The horizontal line running through the square shows the confidence interval of the estimate. The size of each square corresponds to the size of the study and therefore the precision of the estimate. The diamond symbol represents the overall estimate from the meta-analysis, and the horizontal line its confidence interval.

Six studies provided data about the proportion of all ED attendances due to mental or behavioural health disorders. One of these six studies was rated good overall quality;[22] the remainder were assessed to be of fair quality. Pooling this information, we estimates that the proportion of all ED episodes due to mental or behavioural health disorders was 0·04 (95% CI, 0·03–0·04), or 4% (Fig 3). All these six studies examined attendances at hospitals in Australia and one described separate findings from three categories of hospital, which they termed principal referral, major metropolitan and rural (Fig 3). [24] Although one of the studies focused specifically on police presentations to the ED,[28] the authors also included an estimate of the overall proportion of ED attendances that were due to mental health conditions.

### Characteristics of patients presenting to EDs due to mental or behavioural health disorders

#### Clinical reason for attendance

Sixteen studies provided information about the clinical reasons for patients attending the ED. Generally, data on clinical reasons for attendance were reported inconsistently across the sixteen studies, with authors using different methods to classify patients. Using meta-analysis, we were able to estimate proportions of attendances that were due to four specific conditions or problems: suicide attempt/ ideation; self-harm; schizophrenia and depression. In each case, we extracted the number of patients with each condition as described by the study authors; there may be underlying differences in the way that patients were diagnosed and categorised. Meta-analysis was undertaken for these four conditions, because the relevant data were available. However, they represent only some of the reasons why patients may present to an ED. Consequently, pooled percentages do not add up to 100%. In all the studies from which these estimates were derived, data were reported at the level of total ED attendances, rather than individual patients.

Pooling data from three studies, which were all conducted in Australia, we estimates that 9% (0·09, 95% CI 0·05–0·14) of mental health-related attendances were due to a suicide attempt or suicidal ideation (Table 1). Two were assessed to be of fair quality,[20,23] one poor.[21] We conducted a sensitivity analysis removing the poor quality study from the meta-analysis.[21] The pooled proportion estimate reduced slightly as a result (0·08, 95% CI 0·02–0·17). In addition, via meta-analysis, we estimate that approximately 27% of patients who attend the ED because of a mental or behavioural health disorder, do so primarily because of self-harm (0·27, 95% CI 0·21–0·33). These data were pooled from three studies from two countries, Australia (n = 2) and the UK (n = 1). Again, two were of fair quality[22,24] and the third was assessed as poor.[33] The pooled estimated proportion reduced slightly when the poor study[33] was removed from the meta-analysis (0·26, 95% CI 0·20–0·33).

**Table 1:** Meta-analysis: proportion of mental health-related ED attendances due to specific conditions.

	Number of papers	Random effects proportion	95% Confidence Interval	I^2^
**Suicide risk / attempt**	3	0.089	[0.046–0.141]	0%
**Self-harm**	5	0.266	[0.210–0.326]	87.1%
**Schizophrenia**	5	0.055	[0.045–0.066]	0.4%
**Depression**	7	0.134	[0.101–0.170]	76.7%

Similarly, we estimate via meta-analysis that approximately 6% of patients who attend the ED because of mental or behavioural health disorders do so because of schizophrenia (0·06, 95% CI 0·05–0·07), with a further 13% (0·13, 95% CI 0·10–0·17) attending because of depression. Again, all three of the studies in the schizophrenia analysis were conducted in Australia, as were four of the five depression studies. The fifth was conducted in Spain. In the schizophrenia analysis, the three included studies were all assessed to be of fair quality.[20,23,24] One of the five depression studies was of poor quality;[21] two were good[22,29] and the other two fair.[23,24] Removing the poor study from the meta-analysis[21] did not change the estimate.

#### Previous service use or history of mental or behavioural health disorders

Five studies provided information on patients’ past psychiatric history or previous contact with mental health services. In the UK, 58·1% had a previous history of mental illness.[33] This figure was 86·9% in a study of police presentations in Australia.[28] 58·3% of patients aged over 65 attending a psychiatric emergency room in Spain had a history of depressive disorder.[19] In a study of frequent attenders in Ireland, 70·8% had had a prior psychiatric hospital admission.[34] Meanwhile, in Australia, 25·9% of all patients attending for mental health reasons had had a psychiatric admission in the preceding 12 months.[22] In the same study, 36·5% of patients were also current clients of mental health services.[22]

#### Individual patient characteristics

Twelve of the eighteen studies described all mental-health related ED attendances. Only three of these reported patients’ mean age. Two reported that the mean age was 32–33 years.[22,33] The third was limited to patients aged 65 and over; the mean age was 75·3 years.[19] None reported a standard deviation around the mean. Insufficient data were available to enable us to carry out meta-analysis of patient age. Two of these studies were assessed as being of poor overall quality,[19,33] whilst the other was good quality.[22]

With regard to sociodemographic characteristics, data from five studies enabled us to estimate via meta-analysis that 50% of attendances are by women (95% CI 0·45–0·55, *I*^2^ = 7·3%). We rated three of these studies as being of fair quality; the other two were good.

Three included studies provided information about patient ethnicity or country of origin. Two described mental health-related attendances to the same, single ED in Sydney, Australia, at different time periods. One reported that 75% of frequent attendees originally came from English speaking countries.[25] In the second, 69% of all mental health-related attendees came from English speaking countries.[20] The third study also studied attendances in another part of New South Wales, Australia, specifically looking at patients with mental or behavioural health disorders transferred to the hospital by police.[28] 88% of this cohort were Australian; 3% from England; 4% from New Zealand; and 5% from elsewhere. We assessed all three of these studies as being fair quality.

Four studies provided information about patients’ socioeconomic circumstances, but this was reported in different ways. 53% of patients attending an ED in London, UK due to mental or behavioural health disorders, were unemployed[33] in contrast to 83% of frequent attendees at an ED in Galway, Ireland.[34] Also in London, 17% were of no fixed abode,[33] whilst 4% of patients attending EDs in Victoria, Australia, due to mental or behavioural health disorders were resident in crisis accommodation at the time; the same proportion were deemed to have no shelter.[28] 45% of frequent attenders to a dedicated psychiatric ED in Montreal, Canada were in receipt of welfare payments.[31] We assessed three of these four studies as being of fair quality;[28,31,34] one was deemed to be poor.[33]

#### Destination on discharge from the ED

Thirteen studies provided data on patients’ destination on discharge from the ED. Because each study reported this in a different way, we were not able to use meta-analysis to calculate meaningful pooled estimates, for example of the proportion of patients who are admitted to hospital or followed up on an outpatient basis. Considering admission to hospital generally, in Spain, 17% of attendances resulted in admission, but the type of ward was not specified.[29] In an Australian study over half of patients (58%) were admitted.[20] Broken down by type of ward, the proportion of patients admitted to a mental health unit ranged from8%[23] to 27·8%,[19] whilst the proportion admitted to a general medical ward ranged from 6·6%[22] to 16·7%.[19] Similarly, only two studies reported the proportion followed up as an outpatient: 15% of attendances in London resulted in discharge from the ED with GP follow up.[33] In an Australian study of police presentations, 25% of patients required outpatient follow-up by a community mental health team.[28] Six studies reported the proportion of patients discharged home from the ED. This ranged from 36% in a study of all mental health-related attendances,[21] to 67% in study which focused on attendances by patients under section.[26] Both these studies were conducted in Australia. However, within these papers, it was only clear in one case that the discharged patients did not receive any form of follow up.[28] Two Australian studies reported respectively that 6·1%[22] and 8%[21] of mental health-related attendances resulted in the patient leaving the ED without being seen. Again, we assessed these papers as lying across the quality spectrum: four were good,[19,22,26,29] six were fair[20,25,28,31,32,34] and three were poor.[21,30,33]

## Discussion

We identified 18 studies, which together suggest that 4% of ED attendances are made by patients attending because of a mental or behavioural health disorder. A third of these attendances are specifically due to self-harm or suicidal ideation. However, the majority of studies were single site and of low quality so data must be interpreted cautiously. Our estimate is similar to the Medicare figure quoted in current policy (5%).[6] Over half of patients had a past history of psychiatric illness in one study,[33] suggesting that they were ‘known’ to mental health services. In another, a third of patients were in current contact with services.[22] We estimate that half of attendances are made by females, and based on two studies the mean age of patients was 32–33.[22,33] Our findings suggest that a quarter were admitted to a mental health ward, but 6–8% left the ED without waiting to be seen.[21,22] A further third were discharged home from the ED, but it is unclear whether some in this category also received outpatient follow up.

### Study limitations

The data that are available must also be interpreted with caution, in light of issues relating to the quality of the data reported; the overall methodological quality of the studies; and the generalisability of the study findings to other services and local populations. For example, eight studies reported findings in terms of total ED attendances; six in terms of individual patients; and four used both figures, often at different points in the paper. Similarly, where data on past psychiatric history and destination on discharge from the ED were reported, this was done in different ways. This was also the case for data on ethnicity or socioeconomic circumstances. In our analyses, we used data reported in the studies to estimate the proportion of patients attending due to certain conditions. However, studies used a range of different diagnostic methods to classify clinical reasons for attendance. Self-harm or suicidal ideation may represent plausible clinical reasons for presenting to the ED. We suggest, however, that patients with schizophrenia may be more likely to present to an ED with an acute psychosis that is later attributed to mania, schizophrenia, or drug induced psychosis, for example, rather than receiving such a diagnosis in the ED. In addition, only partial information was provided in some cases. For example, regarding destination on discharge from the ED, in some studies data were reported for only a sub-section of the study population, such as the proportion admitted to hospital.

A range of factors also hampered our assessment of the methodological quality of studies, for example limited descriptions of how the study population was identified or how analyses were conducted. Similarly, many papers provided insufficient information about any measures taken to assure the quality of the data, such as accuracy checking, or how data was actually collected in the ED. Many of the included studies also employed different methods of case identification. For example, where this was reported, there were considerable differences between studies in terms of the way patients were identified and categorised. Notably, half of the studies were conducted in Australia and the generalisability of the findings was assessed as poor in 15/18 cases, usually because the study described a relatively small sample from a single hospital site. Over half of the studies were also conducted in urban areas, where the demographic profile is likely to be different, compared to other parts of the country. For example, the prevalence of mental illness is often higher in inner city areas,[36] further reducing the generalisability and relevance of the findings to less densely populated areas.

We sought to reduce heterogeneity between the included studies in our meta-analyses. We also only included papers published since 2000, after the publication of the first National Service Framework for mental health, which redefined care standards in the UK. However, it is likely that there may still be genuine differences underlying the variations in findings by different studies. The most important causes of variability relate to differences in either clinical or methodological aspects of the research.[37] For example, the studies originate from different geographical regions. Consequently, there may be differences in terms of the provision of mental health and emergency services, as well as routes into care. As we have noted, half of the studies examined care in different areas of Australia. Only one was conducted in the UK and this was assessed to be of poor quality.[33] Publication bias seems an unlikely explanation. Search strategies and safeguards against publication bias are less well developed for reviews of observational studies than they are for clinical trials.[38] However, our search strategy was broad and employed both standard terms and procedures.

Finally, there were significant practical advantages to using the text mining function in EPPI-Reviewer 4 to screen 16,000 titles and abstracts, not least because this approach offered a mechanism for truncating the screening process. However, the limitation of this approach is a function of its strength: it expands the review in favour of literature that uses the same language as the documents that have already been found.[10] It does not assist in identifying literatures that use different words to describe the same concepts. For example, although we included a range of possible synonyms in the search strategy, it is feasible that we have missed articles that use different terms to describe hospital emergency services. In addition, because the screening process was truncated, we cannot quantify the number of studies that may have been missed. Finally, ordering the studies in this way may bias the reviewer: they may expect to have more included studies at the beginning of the process, and so be over-inclusive, and likewise, miss studies later in the list because they assume they are looking at less relevant studies.[10] It is possible that we may also have missed relevant studies because our search was only conducted in English. This may also limit the relevance of our findings for non-English speaking countries.

### Implications

Our data show that, although presentations to Emergency Departments due to mental or behavioural health disorders comprise only a small percentage of overall presentations, they are potentially a group with significant morbidity. Between 8% and 27% were admitted to psychiatric in-patient care and 6% to 16% to general medical wards. This suggests a lower range of admission of around 14% and an upper range of over 40%, which contrasts with an admission rate for all Emergency Department attendances of 20·8%. [39] Current best practice in mental health supports the use of community based crisis and home treatment teams as an important means of preventing hospital admissions for people with mental disorders. [40] Given that 58·1% of patients had a previous history of mental illness, and that 36.5% were current clients of mental health services, this raises a question as to whether the community support available to them was inadequate and not meeting their needs. In addition to limited access to crisis teams, it is possible that limited long-term community support may also have played a role in people requiring admission via an Emergency Department. Support for this suggestion comes from evidence of a reduction in mental health funding relative to other areas of health care. [41]

41·9% of patients had no history of mental illness and it is possible that for these patients ED may have been a route into care. Although, this may represent opportunity possibility to correct previously lost opportunities to engage in care in other settings, the fact that over a third of patients (36%) with a mental health diagnosis were sent home directly from the ED, suggests the opportunity to offer mental health care may have been lost again, with needs continuing to be unmet.

Another cause of concern is the large proportion of patients attending the ED due to mental or physical behavioural disorders who present with self-harm or suicidal ideation. While a number of these patients will have had an underlying depressive or psychotic disorders, it is probable that another proportion had an underlying personality disorder. Patients who present with self-harm or suicidal ideation are disproportionately represented in repeat attenders at Emergency Departments [4] and this may reflect a lack of community based service to provide effective care as community services for this group are under-developed

Our review suggests that there is a lack of high quality, generalisable epidemiological data available to inform service change and the development of new models of care. The concerns raised above suggest that limitations in available community based treatments are making a significant demand on Emergency Departments. Further high quality epidemiological studies are needed to inform service improvements and ensure that interventions are targeted appropriately. As a minimum, such studies would involve a large sample of patients, attending a number of different EDs, to maximize the generalisability and validity of the findings. Particular attention would be paid to collecting data, including clinical reason for attendance; past psychiatric history and destination on discharge, to build up a detailed picture of the relevant patient population, using transparent, standardised criteria. Information about patient characteristics such as age; socioeconomic circumstances; and ethnicity would help ensure that services are being targeted to the patient groups that need them most.

## Supporting Information

S1 Appendix Search strategy. (DOCX)

S2 Appendix Scoring system for methodological quality of included studies. (DOCX)

S3 Appendix Overview of included studies (n = 18). (DOCX)

S4 Appendix Summary of main characteristics of included studies (n = 18). (DOCX)

S5 Appendix PRISMA Checklist. (DOC)

S6 Appendix Excluded articles with reasons (n = 186). (DOCX)
