# Supplementary material for: Epidemiology of Mental Health Attendances at Emergency Departments: Systematic Review and Meta-Analysis
Source: PLoS One. 2016 Apr 27;11(4):e0154449. doi: 10.1371/journal.pone.0154449 (PMC4847792; doi:10.1371/journal.pone.0154449)
Supplement: S1 Appendix — (DOCX) [file pone.0154449.s001.docx]

**Appendix S1 Search strategy.**

The search strategy was initially developed for MEDLINE before being translated for use in other databases/interfaces. Strategies were built up through a number of trial searches and discussions of the results with the review team. In order to ensure comprehensive coverage, search terms were kept purposefully broad to help counter dissimilarities in database indexing practices and thesaurus terms, and imprecise reporting of study populations by authors in the text of records.

The strategies for each of the databases searched follow below.

**Embase, Medline, PreMedline, PsycINFO – OVID**

Dates searched: 2000 to 15 July 2014

| 1 | mental disease/ or mental health/ |
| --- | --- |
| 2 | 1 use emez |
| 3 | mental disorders/ use mesz |
| 4 | mental disorders/ or mental health/ |
| 5 | 4 use psyh |
| 6 | ((mental$ or psychologic$) adj2 (health or disorder$ or disease$ or deficien$ or illness or problem$)).ti,ab. or (psychiatric$ or psychologic$).ti. |
| 7 | (or/2-3,5-6) or complex psychological.ti,ab. |
| 8 | cognitive defect/ or exp dementia/ |
| 9 | 8 use emez |
| 10 | cognition disorders/ or exp dementia/ |
| 11 | 10 use mesz |
| 12 | alzheimer's disease/ or cognitive impairment/ or creutzfeldt jakob syndrome/ or exp dementia/ or picks disease/ |
| 13 | 12 use psyh |
| 14 | (alzheim$ or binswanger$ or cadasil or cerad or dement$ or ftld or ftd$ or ((frontotemporal or fronto temporal or corticobasal or cortico basal or frontolob$ or fronto lob$) adj5 (degenerat$4 or dysfunction$)) or (kluver adj5 (bucy or busy)) or (lew$2 adj5 bod$3) or dlbd or (lobar adj5 atroph$3 adj5 (brain or cerebr$2)) or (mesulam adj5 syndrome$) or (pick$2 adj5 (disease$1 or complex)) or posterior corticotroph$ or posterior corticotroph$ or ((primary or progressive) adj5 aphasi$) or sdat or sivd or ((subcortic$3 or sub cortic$3) adj5 (encephalopath$3 or leukoencephalopath$3))).ti,ab. |
| 15 | or/9,11,13-14 |
| 16 | addiction or exp alcohol abuse/ or exp detoxification or exp drug dependence/ or exp drug abuse/ or substance abuse/ |
| 17 | 16 use emez |
| 18 | behavior addictive/ or drug seeking behavior/ or exp alcohol-related disorders/ or amphetamine-related disorders/ or cocaine-related disorders/ or drug overdose/ or inhalant abuse/ or marijuana abuse/ or exp opioid-related disorders/ or phencyclidine abuse/ or psychoses, substance-induced/ or substance abuse, intravenous/ or substance-related disorders/ or exp substance withdrawal syndrome/ |
| 19 | 18 use mesz |
| 20 | addiction or exp alcoholism/ or drug abuse prevention or exp drug addiction or exp drug abuse/ or sobriety/ |
| 21 | 20 use psyh |
| 22 | (needle adj (exchange or sharing)).sh. |
| 23 | (alcoholi$ or drinker$1 or (drink$ adj2 use$1) or ((alcohol$ or drink$) adj5 (abstinen$ or abstain$ or abus$ or addict$ or attenuat$ or bing$ or crav$ or dependen$ or detox$ or disease$ or disorder$ or excessiv$ or harm$ or hazard$ or heavy or high risk or intoxicat$ or misus$ or overdos$ or (over adj dos$) or problem$ or rehab$ or reliance or reliant or relaps$ or withdraw$)) or (control$ adj2 drink$) or sobriet$).ti,ab. |
| 24 | (((acetomorphine or amphetamine$ or amphetamine$ or analeptic$ or cannabis or cocaine or crack or crank or dextroamphetamine$ or diacephine or diacetylmorphine or diacetylmorphine or diamorphin$ or diamorphine or diaphorin or drug or hashish or heroin or marihuana or marijua$ or methadone$ or methamphetamine$ or morfin$ or morphacetin or morphin$ or naltrexone or narcotic$ or opioid$ or opium or polydrug$ or psychostimulant$ or speed or stimulant$ or stimulant$ or substance or uppers or cigarette$ or nicotin$ or smoking or tobacco) adj3 (abstain$ or abstinen$ or abus$ or addict$ or (excessive adj use$) or dependen$ or (inject$ adj2 drug$) or intoxicat$ or misus$ or over dos$ or overdos$ or (use$ adj (disorder$ or illicit)) or withdraw$)) or drug user$).ti,ab. |
| 25 | or/17,19,21-24 |
| 26 | automutilation or self poisoning/ or suicide/ or suicide attempt/ or suicidal ideation |
| 27 | 26 use emez |
| 28 | self-injurious behavior/ or self mutilation or suicide/ or suicidal ideation or suicide, attempted/ |
| 29 | 28 use mesz |
| 30 | suicide/ or attempted suicide/ or exp self injurious behavior/ or suicidal ideation or suicide prevention or suicidology/ |
| 31 | 30 use psyh |
| 32 | (autoaggress$ or auto aggress$ or automutilat$ or auto mutilat$ or cutt$ or overdos$ or over dos$ or (self adj2 cut$) or selfdestruct$ or self destruct$ or selfharm$ or self harm$ or selfimmolat$ or self immolat$ or selfinflict$ or self inflict$ or selfinjur$ or self injur$ or selfmutilat$ or self mutilat$ or selfpoison$ or self poison$ or suicid$).ti,ab. |
| 33 | or/27,29,31-32 |
| 34 | exp psychosis/ |
| 35 | 34 use emez |
| 36 | exp "schizophrenia and disorders with psychotic features"/ |
| 37 | 36 use mesz |
| 38 | paranoid schizophrenia/ or exp psychosis/ or schizoaffective disorder/ |
| 39 | 38 use psyh |
| 40 | (delusion$ or hallucinat$ or hebephreni$ or oligophreni$ or paranoi$ or psychotic$ or psychosis or psychoses or schizo$).ti,ab. |
| 41 | or/35,37,39-40 |
| 42 | exp mood disorder/ |
| 43 | 42 use emez |
| 44 | exp mood disorders/ |
| 45 | 44 use mesz |
| 46 | exp affective disorders/ |
| 47 | 46 use psyh |
| 48 | ((affective or mood) adj2 (disorder$ or dysfunction$ or psychosis$)).ti,ab. |
| 49 | ( ((bipolar or bi polar) adj5 (disorder$ or depress$)) or ((cyclothymi$ or rapid or ultradian) adj5 cycl$) or hypomani$ or mania$ or manic$ or mixed episode$ or rcbd).ti,ab. |
| 50 | (depres$ or dysthym$ or melancholi$).ti,ab. |
| 51 | or/43,45,47-50 |
| 52 | anxiety/ or anxiety disorder/ or anxiety neurosis/ or generalized anxiety disorder/ or "mixed anxiety and depression"/ |
| 53 | 52 use emez |
| 54 | anxiety/ or anxiety disorders/ |
| 55 | 54 use mesz |
| 56 | anxiety/ or anxiety disorders/ or generalized anxiety disorder/ |
| 57 | 56 use psyh |
| 58 | anxiety.sh. or (anxiet$ or ((chronic$ or excessiv$ or intens$ or (long$ adj2 last$) or neuros$ or neurotic$ or ongoing or persist$ or serious$ or sever$ or uncontrol$ or un control$ or unrelent$ or un relent$) adj2 (anxious$ or worry))).ti,ab. |
| 59 | or/53,55,57-58 |
| 60 | hysteria/ or exp somatoform disorder/ |
| 61 | 60 use emez |
| 62 | hysteria/ or exp somatoform disorders/ |
| 63 | 62 use mesz |
| 64 | hysteria/ or exp somatoform disorders/ or somatization |
| 65 | 64 use psyh |
| 66 | (briquet or hysteri$ or polysymptom$ or poly symptom$ or multisomat$ or multi somat$ or (multiple and (mups or medically unexplained or unexplained symptoms or physical symptoms or symptom diagnos$)) or somatiz$ or somatis$).ti,ab. |
| 67 | or/61,63,65-66 |
| 68 | anorexia/ or appetite disorder/ or exp eating disorder/ |
| 69 | 68 use emez |
| 70 | anorexia/ or bulimia/ or exp eating disorders/ |
| 71 | 70 use mesz |
| 72 | binge eating/ or exp eating disorders/ |
| 73 | 72 use psyh |
| 74 | (anorexi$ or bulimi$ or ((bing$ or compulsive$) adj2 (eat$ or vomit$)) or (eating adj2 disorder$) or (food$ adj2 bing$) or overeat$ or over$1 eat$ or (restrict$ adj2 eat$) or ((self induc$ or selfinduc$) adj2 vomit$) or (((abnormal$ or disturbance$ or disturbed or dysfunction$ or problem$) adj2 eating) or anorectic or ((avoidant adj2 restrictive food intake disorder) or arfid) or (bing$ adj2 (episode$ or meal$)) or (eating adj (pathology or psychopathology)) or emotional eating or ((eat or food) adj2 addict$) or loss of control eating or orthorexi$ or pica)).ti,ab. |
| 75 | or/69,71,73-74 |
| 76 | borderline state/ or exp personality disorder/ |
| 77 | 76 use emez |
| 78 | exp personality disorders/ |
| 79 | 78 use mesz |
| 80 | borderine states/ or exp personality disorders/ |
| 81 | 80 use psyh |
| 82 | character disorder.sh. |
| 83 | (((antisocial or anti social or avoidant or borderline or dependent or histrionic or narcissistic or paranoid or passive aggressive or schizotypal) adj3 (disorder$ or person$ or pd$1 or state$)) or (emotion$ adj2 (instabil$ or unstable) adj3 (character$ or difficult$ or disorder$ or dysfunction$ or pd or person$1 or personalit$ or state$)) or (personalit$ adj2 (disorder$ or dysfunction$)) or (borderline$ and cluster b) or (dsm and (axis and ii)) or narcissism or psychopathy or schizoid$).ti,ab. |
| 84 | or/77,79,81-83 |
| 85 | exp developmental disorder/ or intellectual impairment/ or exp mental deficiency/ or exp learning disorder/ |
| 86 | 85 use emez |
| 87 | developmental disabilities/ or exp intellectual disability/ or exp learning disorders/ or mentally disabled persons/ |
| 88 | 87 use mesz |
| 89 | exp developmental disabilities/ or exp intellectual development disorder/ or exp learning disabilities/ |
| 90 | 89 use psyh |
| 91 | ((intellect$ adj (deficien$ or difficult$ or disab$ or disorder$ or impair$ or incapacit$ or subnorm$ or sub norm$ or subaverage or sub average)) or (low$2 adj2 intellect$) or (learning adj (deficien$ or difficult$ or disab$ or disorder$ or impair$ or incapacit$ or subnorm$ or sub norm$ or subaverage or sub average)) or (mental$ adj (deficien$ or disab$ or impair$ or incapacit$ or retard$ or subnorm$ or sub norm$ or subaverage or sub average )) or handicap$).ti,ab. |
| 92 | (((subaverage or sub average or subnormal or sub normal) adj3 (cognit$ or intel$)) or ((development$ or neurodevelopment$) adj disab$) or (cretin$ or feeble minded$ or imbecil$ or moron$) or (multiple disabilit$ or developmental delay$)).ti,ab. |
| 93 | down syndrome/ |
| 94 | 93 use emez |
| 95 | angelman syndrome/ or down syndrome/ or fragile x syndrome/ |
| 96 | 95 use mesz |
| 97 | down's syndrome/ |
| 98 | 97 use psyh |
| 99 | (((angelman or happy puppet) adj2 syndrom$) or (down$1 adj (disease or syndrome$)) or bell martin or fragile x or gillian turner or martin bell or turner gillian or x chromosome fragility or (escalante$ adj (disease or syndrome$))).ti,ab. |
| 100 | or/86,88,90-92,94,96,98-99 |
| 101 | or/7,15,25,33,41,51,59,67,75,84,100 |
| 102 | emergency health service/ use emez or emergency ward/ use emez or exp emergency service, hospital/ use mesz or emergency services/ use psyh |
| 103 | (a&e or (accident adj2 emergenc$) or casualty or (emergenc$ adj2 (admit$ or admission$ or attendanc$ or center$ or centre$ or department$ or hospital$ or medicine or patient$ or present$ or room$ or unit$ or visit$ or ward$)) or (ed adj (admit$ or admission$ or attendanc$ or patient$ or presentation$ or room$ or service$)) or hospital ed or resus or (trauma adj2 (center$ or centre$ or department$ or unit$ or ward$))).ti,ab. |
| 104 | or/102-103 |
| 105 | exp emergency care/ or emergency medicine/ or emergency nurse/ or emergency physician/ or emergency treatment/ or emergency/ |
| 106 | 105 use emez |
| 107 | emergencies/ or emergency nursing/ or emergency medical services/ or emergency medicine/ or emergency services, psychiatric/ or emergency treatment/ |
| 108 | 107 use mesz |
| 109 | emergency management/ |
| 110 | 109 use psyh |
| 111 | ((acute adj2 (care or manag$ or treat$)) or ((emergenc$ or urgent$) adj3 (admit$ or admission$ or attendanc$ or care or centre$ or center$ or department$ or healthcare or hospital$ or manag$ or medicine or patient$ or present$ or room$ or service$ or setting$ or treat$ or unit$ or visit$ or ward$ or wing)) or (emergenc$ adj2 (doctor$ or nurs$ or physician$)) or ((unscheduled or un scheduled or unplanned or un planned) adj care)).ti,ab. or (triage and (emergen$ or urgent$)).ti,ab,hw. |
| 112 | (or/106,108,110-111) and hospital$.ti,ab,hw. |
| 113 | 104 or 112 |
| 114 | epidemiology.sh,tw. or epidemiological data.sh. or ep.fs. |
| 115 | incidence$.tw,sh. |
| 116 | occurrence$.tw. |
| 117 | prevalen$.tw,sh. |
| 118 | morbidit$.tw,sh. |
| 119 | or/114-118 |
| 120 | (frequen$ or number$ or percent$ or population$ or rate$).tw. or mortalit$.sh,tw. |
| 121 | 119 or 120 |
| 122 | 101 and 113 and 121 |
| 123 | limit 122 to english language |
| 124 | limit 123 to yr="2000 -current" |
|  |  |

**CINAHL – Ebsco**

Dates searched: 2000 to 15 July 2014

Top of Form

| s86 | s60 and s74 and s85 published date: 20000101-2014; english language |
| --- | --- |
| s85 | s81 or s84 |
| s84 | s82 or s83 |
| s83 | mw mortalit* or tx mortalit* |
| s82 | ti ( (frequen* or number* or percent* or population* or rate*) ) or ab ( (frequen* or number* or percent* or population* or rate*) ) |
| s81 | s75 or s76 or s77 or s78 or s79 or s80 |
| s80 | tx morbidit* or mw morbidit* |
| s79 | tx prevalen* or mw prevalen* |
| s78 | ti occurrence* or ab occurrence* |
| s77 | tx incidence* or mw incidence* |
| s76 | (mh "epidemiological research") |
| s75 | (mh "epidemiology") |
| s74 | s63 or s73 |
| s73 | s71 and s72 |
| s72 | tx hospital* or mw hospital* |
| s71 | s64 or s65 or s66 or s67 or s68 or s69 or s70 |
| s70 | tx ( (triage and (emergen* or urgent*)) ) or mw ( (triage and (emergen* or urgent*)) ) |
| s69 | ti ( ((acute n2 (care or manag* or treat*)) or ((emergenc* or urgent*) n3 (admit* or admission* or attendanc* or care or centre* or center* or department* or healthcare or hospital* or manag* or medicine or patient* or present* or room* or service* or setting* or treat* or unit* or visit* or ward* or wing)) or (emergenc* n2 (doctor* or nurs* or physician*)) or ((unscheduled or “un scheduled” or unplanned or “un planned”) n1 care)) ) or ab ( ((acute n2 (care or manag* or treat*)) or ((emergenc* or urgent*) n3 (admit* or admission* or attendanc* or care or centre* or center* or department* or healthcare or hospital* or manag* or medicine or patient* or present* or room* or service* or setting* or treat* or unit* or visit* or ward* or wing)) or (emergenc* n2 (doctor* or nurs* or physician*)) or ((unscheduled or “un scheduled” or unplanned or “un planned”) n1 care)) ) |
| s68 | (mh "emergency treatment (non-cinahl)") or (mh "emergency medical treatment and active labor act") or (mh "emergency patients") |
| s67 | (mh "emergency medicine") or (mh "psychiatric emergencies") |
| s66 | (mh "emergency medical services") or (mh "emergency services, psychiatric") |
| s65 | (mh "emergency nursing") or (mh "trauma nursing") |
| s64 | (mh "emergencies") or (mh "emergency nurse practitioners") or (mh "physicians, emergency") |
| s63 | s61 or s62 |
| s62 | ti ( (a&e or (accident n2 emergenc*) or casualty or (emergenc* n2 (admit* or admission* or attendanc* or center* or centre* or department* or hospital* or medicine or patient* or present* or room* or unit* or visit* or ward*)) or (ed n1 (admit* or admission* or attendanc* or patient* or presentation* or room* or service*)) or hospitalised or hospitalized or resus or (trauma n2 (center* or centre* or department* or unit* or ward*))) ) or ab ( (a&e or (accident n2 emergenc*) or casualty or (emergenc* n2 (admit* or admission* or attendanc* or center* or centre* or department* or hospital* or medicine or patient* or present* or room* or unit* or visit* or ward*)) or (ed n1 (admit* or admission* or attendanc* or patient* or presentation* or room* or service*)) or hospitalised or hospitalized or resus or (trauma n2 (center* or centre* or department* or unit* or ward*))) ) |
| s61 | (mh "emergency service") |
| s60 | s1 or s2 or s3 or s7 or s18 or s25 or s28 or s34 or s37 or s41 or s44 or s48 or s59 |
| s59 | s49 or s50 or s51 or s52 or s53 or s54 or s55 or s56 or s57 or s58 |
| s58 | ti ( (((angelman or “happy puppet”) n2 syndrom*) or (down* n1 (disease or syndrome*)) or “bell martin” or “fragile x” or “gillian turner” or “martin bell” or “turner gillian” or “x chromosome fragility” or (escalante* n1 (disease or syndrome*))) ) or ab ( (((angelman or “happy puppet”) n2 syndrom*) or (down* n1 (disease or syndrome*)) or “bell martin” or “fragile x” or “gillian turner” or “martin bell” or “turner gillian” or “x chromosome fragility” or (escalante* n1 (disease or syndrome*))) ) |
| s57 | (mh "fragile x syndrome") |
| s56 | (mh "down syndrome") |
| s55 | (mh "mentally disabled persons") or (mh "angelman syndrome") |
| s54 | ti ( (((subaverage or “sub* average” or subnormal or “sub* normal*”) n3 (cognit* or intel*)) or ((development* or neurodevelopment*) n1 disab*) or (cretin* or “feeble minded*” or imbecil* or moron*) or ( “multiple disabilit*” or “developmental delay*”)) ) or ab ( (((subaverage or “sub* average” or subnormal or “sub* normal*”) n3 (cognit* or intel*)) or ((development* or neurodevelopment*) n1 disab*) or (cretin* or “feeble minded*” or imbecil* or moron*) or ( “multiple disabilit*” or “developmental delay*”)) ) |
| s53 | ti ( ((intellect* n1 (deficien* or difficult* or disab* or disorder* or impair* or incapacit* or subnorm* or subaverage or “sub* average” or “sub* norm*”)) or (low* n2 intellect*) or (learning n1 (deficien* or difficult* or disab* or disorder* or impair* or incapacit* or subnorm* or “sub* average” or “subaverage” or “sub* norm*”)) or (mental* n1 (deficien* or disab* or impair* or incapacit* or retard* or subnorm* or “subaverage” or “sub* average” or “sub* norm*”))) or handicap*) or ab ( ((intellect* n1 (deficien* or difficult* or disab* or disorder* or impair* or incapacit* or subnorm* or subaverage or “sub* average” or “sub* norm*”)) or (low* n2 intellect*) or (learning n1 (deficien* or difficult* or disab* or disorder* or impair* or incapacit* or subnorm* or “sub* average” or “subaverage” or “sub* norm*”)) or (mental* n1 (deficien* or disab* or impair* or incapacit* or retard* or subnorm* or “subaverage” or “sub* average” or “sub* norm*”))) or handicap* ) |
| s52 | (mh "mentally disabled persons") |
| s51 | (mh "learning disorders+") |
| s50 | (mh "intellectual disability+") |
| s49 | (mh "developmental disabilities") |
| s48 | s45 or s46 or s47 |
| s47 | ti ( emotion* and (instabil* or unstable) and (character* or difficult* or disorder* or dysfunction* or pd or person* or personalit* or state*) ) or ab ( emotion* and (instabil* or unstable) and (character* or difficult* or disorder* or dysfunction* or pd or person* or personalit* or state*) ) |
| s46 | ti ( ( (((antisocial or “anti social” or avoidant or borderline or dependent or histrionic or narcissistic or paranoid or “passive aggressive” or schizotypal) n3 (disorder* or person* or pd* or state*)) or (personalit* n2 (disorder* or dysfunction*)) or (borderline* and “cluster b”) or (dsm and (axis and ii)) or narcissism or psychopathy or schizoid*) ) ) or ab ( ( (((antisocial or “anti social” or avoidant or borderline or dependent or histrionic or narcissistic or paranoid or “passive aggressive” or schizotypal) n3 (disorder* or person* or pd* or state*)) or (personalit* n2 (disorder* or dysfunction*)) or (borderline* and “cluster b”) or (dsm and (axis and ii)) or narcissism or psychopathy or schizoid*) ) ) |
| s45 | (mh "personality disorders+") |
| s44 | s42 or s43 |
| s43 | ti (anorexi* or bulimi* or ((bing* or compulsive*) n2 (eat* or vomit*))  or (eating n2 disorder*) or (food* n2 bing*) or overeat* or "over* eat*"  or (restrict* n2 eat*) or (("self induc*" or selfinduc*) n2 vomit*) or  (((abnormal* or disturbance* or disturbed or dysfunction* or problem*) n2 eating)  or anorectic or ((avoidant n2 "restrictive food intake disorder") or arfid) or  (bing* n2 (episode* or meal*)) or (eating n1 (pathology or psychopathology))  or "emotional eating" or ((eat or food) n2 addict*) or "loss of control eating"  or orthorexi* or pica)) or ab (anorexi* or bulimi* or ((bing* or compulsive*) n2 (eat* or vomit*)) or (eating n2 disorder*) or (food* n2 bing*) or overeat* or "over* eat*"  or (restrict* n2 eat*) or (("self induc*" or selfinduc*) n2 vomit*) or  (((abnormal* or disturbance* or disturbed or dysfunction* or problem*) n2 eating)  or anorectic or ((avoidant n2 "restrictive food intake disorder") or arfid) or  (bing* n2 (episode* or meal*)) or (eating n1 (pathology or psychopathology))  or "emotional eating" or ((eat or food) n2 addict*) or "loss of control eating"  or orthorexi* or pica)) |
| s42 | (mh "eating disorders+") or (mh "eating disorders management (iowa nic)") |
| s41 | s38 or s39 or s40 |
| s40 | ti ( (briquet or hysteri* or polysymptom* or “poly symptom*” or multisomat* or “multi somat*” or (multiple and (mups or “medically unexplained” or “unexplained symptoms” or “physical symptoms” or “symptom diagnos*”)) or somatiz* or somatis*) ) or ab ( (briquet or hysteri* or polysymptom* or “poly symptom*” or multisomat* or “multi somat*” or (multiple and (mups or “medically unexplained” or “unexplained symptoms” or “physical symptoms” or “symptom diagnos*”)) or somatiz* or somatis*) ) |
| s39 | (mh "somatoform disorders+") |
| s38 | (mh "hysteria") |
| s37 | s35 or s36 |
| s36 | ti ( (anxiet* or anxious* or ((chronic* or excessiv* or intens* or (long* n2 last*) or neuros* or neurotic* or ongoing or persist* or serious* or sever* or uncontrol* or “un control*” or unrelent* or “un relent*”) n2 worry)) ) or ab ( (anxiet* or anxious* or ((chronic* or excessiv* or intens* or (long* n2 last*) or neuros* or neurotic* or ongoing or persist* or serious* or sever* or uncontrol* or “un control*” or unrelent* or “un relent*”) n2 worry)) ) |
| s35 | (mh "anxiety") or (mh "generalized anxiety disorder") or (mh "anxiety (saba ccc)") or (mh "anxiety disorders") |
| s34 | s29 or s30 or s31 or s32 or s33 |
| s33 | ti ( (depres* or dysthym* or melancholi*) ) or ab ( (depres* or dysthym* or melancholi*) ) |
| s32 | ti ( (((bipolar or “bi polar”) n5 (disorder* or depress*)) or ((cyclothymi* or rapid or ultradian) n5 cycl*) or hypomani* or mania* or manic* or “mixed episode*” or rcbd) ) or ab ( (((bipolar or “bi polar”) n5 (disorder* or depress*)) or ((cyclothymi* or rapid or ultradian) n5 cycl*) or hypomani* or mania* or manic* or “mixed episode*” or rcbd) ) |
| s31 | ti ( ((affective or mood) n2 (disorder* or dysfunction* or psychosis*)) ) or ab ( ((affective or mood) n2 (disorder* or dysfunction* or psychosis*)) ) |
| s30 | (mh "affective disorders, psychotic+") |
| s29 | (mh "affective disorders") or (mh "depression+") |
| s28 | s26 or s27 |
| s27 | ti ( (delusion* or hallucinat* or hebephreni* or oligophreni* or paranoi* or psychotic* or psychosis or psychoses or schizo*) ) or ab ( (delusion* or hallucinat* or hebephreni* or oligophreni* or paranoi* or psychotic* or psychosis or psychoses or schizo*) ) |
| s26 | (mh "psychotic disorders+") |
| s25 | s19 or s20 or s21 or s22 or s23 or s24 |
| s24 | ti ( (autoaggress* or “auto aggress*” or automutilat* or “auto mutilat*” or cutt* or overdose* or (self n2 cut*) or selfdestruct* or “self destruct*” or selfharm* or “self harm*” or selfimmolat* or “self immolat*” or selfinflict* or “self inflict*” or selfinjur* or “self injur*” or selfmutilat* or “self mutilat*” or selfpoison* or “self poison*” or suicid*) ) or ab ( (autoaggress* or “auto aggress*” or automutilat* or “auto mutilat*” or cutt* or overdose* or (self n2 cut*) or selfdestruct* or “self destruct*” or selfharm* or “self harm*” or selfimmolat* or “self immolat*” or selfinflict* or “self inflict*” or selfinjur* or “self injur*” or selfmutilat* or “self mutilat*” or selfpoison* or “self poison*” or suicid*) ) |
| s23 | (mh "suicide") or (mh "suicidal ideation") or (mh "suicide, assisted") or (mh "suicide, attempted") or (mh "suicide risk (saba ccc)") or (mh "suicide self-restraint (iowa noc)") or (mh "suicide prevention (iowa nic)") |
| s22 | (mh "suicide") or (mh "suicidal ideation") or (mh "suicide, assisted") or (mh "suicide, attempted") |
| s21 | (mh "injuries, self-inflicted") |
| s20 | (mh "self-mutilation restraint (iowa noc)") or (mh "abusive behavior self-control (iowa noc)") |
| s19 | (mh "self-injurious behavior") |
| s18 | s8 or s9 or s10 or s11 or s12 or s13 or s14 or s15 or s16 or s17 |
| s17 | ti ( (((acetomorphine or amphetamine* or amphetamine* or analeptic* or cannabis or cocaine or crack or crank or dextroamphetamine* or diacephine or diacetylmorphine or diacetylmorphine or diamorphin* or diamorphine or diaphorin or drug or hashish or heroin or marihuana or marijua* or methadone* or methamphetamine* or morfin* or morphacetin or morphin* or naltrexone or narcotic* or opioid* or opium or polydrug* or psychostimulant* or speed or stimulant* or stimulant* or substance or uppers or cigarette* or nicotin* or smoking or tobacco) n3 (abstain* or abstinen* or abus* or addict* or (excessive n1 use*) or dependen* or (inject* n2 drug*) or intoxicat* or misus* or “over dos*” or overdos* or (use*1 n1 (disorder* or illicit)) or withdraw*)) or “drug user*”) ) or ab ( (((acetomorphine or amphetamine* or amphetamine* or analeptic* or cannabis or cocaine or crack or crank or dextroamphetamine* or diacephine or diacetylmorphine or diacetylmorphine or diamorphin* or diamorphine or diaphorin or drug or hashish or heroin or marihuana or marijua* or methadone* or methamphetamine* or morfin* or morphacetin or morphin* or naltrexone or narcotic* or opioid* or opium or polydrug* or psychostimulant* or speed or stimulant* or stimulant* or substance or uppers or cigarette* or nicotin* or smoking or tobacco) n3 (abstain* or abstinen* or abus* or addict* or (excessive n1 use*) or dependen* or (inject* n2 drug*) or intoxicat* or misus* or “over dos*” or overdos* or (use*1 n1 (disorder* or illicit)) or withdraw*)) or “drug user*”) ) |
| s16 | ti ( (alcoholi* or drinker* or (drink* n2 use* ) or ((alcohol* or drink*) n5 (abstinen* or abstain* or abus* or addict* or attenuat* or bing* or crav* or dependen* or detox* or disease* or disorder* or excessiv* or harm* or hazard* or heavy or “high risk” or intoxicat* or misus* or overdos* or (over n1 dos*) or problem* or rehab* or reliance or reliant or relaps* or withdraw*)) or (control* n2 drink*) or sobriet*) ) or ab ( (alcoholi* or drinker* or (drink* n2 use* ) or ((alcohol* or drink*) n5 (abstinen* or abstain* or abus* or addict* or attenuat* or bing* or crav* or dependen* or detox* or disease* or disorder* or excessiv* or harm* or hazard* or heavy or “high risk” or intoxicat* or misus* or overdos* or (over n1 dos*) or problem* or rehab* or reliance or reliant or relaps* or withdraw*)) or (control* n2 drink*) or sobriet*) ) |
| s15 | (mh "needle exchange programs") |
| s14 | (mh "substance withdrawal syndrome") or (mh "alcohol withdrawal syndrome") or (mh "neonatal abstinence syndrome") or (mh "organic mental disorders, substance-induced") |
| s13 | (mh "psychoses, substance-induced+") |
| s12 | (mh "substance abusers+") or (mh "substance abuse detection+") |
| s11 | (mh "substance abusers+") |
| s10 | (mh "substance use treatment (iowa nic)+") |
| s9 | (mh "behavior, addictive") |
| s8 | (mh "substance abuse+") |
| s7 | s4 or s5 or s6 |
| s6 | ti ( (alzheim* or binswanger* or cadasil or cerad or dement* or ftld or ftd* or ((frontotemporal or “fronto temporal” or corticobasal or “cortico basal” or “frontal lobe”) n5 (degenerat* or dysfunction*)) or (kluver n5 (bucy or busy)) or (lew* n5 bod*) or dlbd or (lobar n5 atroph* n5 (brain or cerebr*2)) or (mesulam n5 syndrome*) or (pick* n5 (disease* or complex)) or “posterior cortic* atroph*” or ((primary or progressive) n5 aphasi*) or sdat or sivd or ((subcortic* or “sub cortic*”) n5 (encephalopath* or leukoencephalopath*))) ) or ab ( (alzheim* or binswanger* or cadasil or cerad or dement* or ftld or ftd* or ((frontotemporal or “fronto temporal” or corticobasal or “cortico basal” or “frontal lobe”) n5 (degenerat* or dysfunction*)) or (kluver n5 (bucy or busy)) or (lew* n5 bod*) or dlbd or (lobar n5 atroph* n5 (brain or cerebr*2)) or (mesulam n5 syndrome*) or (pick* n5 (disease* or complex)) or “posterior cortic* atroph*” or ((primary or progressive) n5 aphasi*) or sdat or sivd or ((subcortic* or “sub cortic*”) n5 (encephalopath* or leukoencephalopath*))) ) |
| s5 | (mh "cognition disorders") |
| s4 | (mh "dementia+") |
| s3 | ti " complex psychological" or ab " complex psychological" |
| s2 | ti ( ((mental* or psychologic*) n2 (health or disorder* or disease* or deficien* or illness or problem*)) ) or ab ( ((mental* or psychologic*) n2 (health or disorder* or disease* or deficien* or illness or problem*)) ) or ( (psychiatric* or psychologic*) ) |
| s1 | (mh "mental disorders") |
