# Supplementary material for: Epidemiology of Mental Health Attendances at Emergency Departments: Systematic Review and Meta-Analysis
Source: PLoS One. 2016 Apr 27;11(4):e0154449. doi: 10.1371/journal.pone.0154449 (PMC4847792; doi:10.1371/journal.pone.0154449)
Supplement: S2 Appendix — (DOCX) [file pone.0154449.s002.docx]

**Appendix S2 Scoring system for methodological quality of included studies.**

| 1 | **Did the study address a clearly focused issue?**   - *A question can be focused in terms of:*  1. *the population(s) studied* 2. *the epidemiological variables studied*  - *Did the authors describe their goal in conducting this research?* - *Is it easy to understand what they were looking to find?* | - Good - Fair - Poor - Can’t tell |
| --- | --- | --- |
| 2 | **Did the authors use an appropriate method to answer their question?**  *Consider*   - *Is a descriptive/cross-sectional study an appropriate way of answering the question?* - *Did it address the study question?* | - Good - Fair - Poor - Can’t tell |
| 3 | **Was the study population clearly specified and defined?**   - *Did the authors describe the group of people from which the study population was selected?* - *If you were to conduct this study again, would you know which patients to include?* | - Good - Fair - Poor - Can’t tell |
| 4 | **Were measures taken to accurately reduce measurement bias?**  *Consider whether measurement bias might compromise the findings:*   - Were variables defined in detail? - Were the tools or methods used to measure relevant variables accurate and reliable–for example, have they been validated or are they objective? - *Did they use subjective or objective measurements?* - *Do the measures truly reflect what you want them to (have they been validated)?* | - Good - Fair - Poor - Can’t tell |
| 5 | **Were the study data collected in a way that addressed the research issue?**  *Consider:*   - *if the setting for data collection was justified* - *if it is clear how data were collected (e.g., interview, questionnaire, chart review)* - *if the researcher has justified the methods chosen* - *if the researcher has made the methods explicit*   *(e.g. for interview method, is there an indication of how interviews were conducted?)* | - Good - Fair - Poor - Can’t tell |
| 6 | **Did the study have enough participants to minimize the play of chance?**  *Consider:*   - *if the result is precise enough to make a decision* - *if there is a power calculation. This will estimate how many subjects are needed to produce a reliable estimate of the measure(s) of interest.* | - Good - Fair - Poor - Can’t tell |
| 7 | **Did the authors take sufficient steps to assure the quality of the study data?**  *For example, did they:*   - *Use standardised data extraction tools* - *Employ double data extraction methods* - *Double check a sample of notes to confirm the accuracy of data collection?* - *Confirm that consecutive patients were included?* | - Good - Fair - Poor - Can’t tell |
| 8 | **Was the data analysis sufficiently rigorous?**  *Consider:*   - *if there is an in-depth description of the analysis process* - *if sufficient data are presented to support the findings* | - Good - Fair - Poor - Can’t tell |
| 9 | **How complete is the discussion?**  *Consider:*   - *Is there a clear statement of findings?* - *Do the authors discuss the limitations of their study?* - *Do the authors set their findings in the context of other studies?* | - Good - Fair - Poor - Can’t tell |
| 10 | **To what extent are the findings generalizable to other international contexts?**  *Consider:*   - *Number of study sites* - *Type of EDs included* - *Size of study population* - *Length of study period*   *Consider also whether selection bias which might compromise the generalizability of the findings:*  *- Was the study sample representative of the patient population?*  *- Was everybody included who should have been included?* | - Good - Fair - Poor - Can’t tell |
|  | **Your overall judgement of the paper**  *(Please provide an overall judgement about the quality of the paper, taking into account your responses to questions 50-59)* | - Good - Fair - Poor   Can’t tell |
