# Supplementary material for: Epidemiology of Mental Health Attendances at Emergency Departments: Systematic Review and Meta-Analysis
Source: PLoS One. 2016 Apr 27;11(4):e0154449. doi: 10.1371/journal.pone.0154449 (PMC4847792; doi:10.1371/journal.pone.0154449)
Supplement: S3 Appendix — (DOCX) [file pone.0154449.s003.docx]

**Appendix 3 Overview of included studies (n=18).**

|  | | N | % |  |
| --- | --- | --- | --- | --- |
| *Study Setting* | **Country in which study was conducted** |  |  |  |
|  | UK | 1 | 6 |  |
|  | Australia | 9 | 50 |  |
|  | Ireland | 1 | 6 |  |
|  | Norway | 1 | 6 |  |
|  | Spain | 3 | 17 |  |
|  | Canada | 2 | 11 |  |
|  | Portugal | 1 | 6 |  |
|  | **Setting** |  |  |  |
|  | General Emergency Department | 13 | 72 |  |
|  | Dedicated Psychiatry Emergency Department | 5 | 28 |  |
|  | **Number of study sites** |  |  |  |
|  | 1 | 14 | 78 |  |
|  | 2 | 2 | 11 |  |
|  | >=3 | 2 | 11 |  |
|  | **Urbanisation** |  |  |  |
|  | Rural | 0 | 0 |  |
|  | Urban | 10 | 56 |  |
|  | Suburban | 3 | 17 |  |
|  | Mixed Urban, Suburban and Rural | 3 | 17 |  |
|  | Other | 1 | 6 |  |
|  | Not reported | 1 | 6 |  |
| *Study Design* | **Study design** |  |  |  |
|  | *Cross-sectional design* |  |  |  |
|  | Retrospective | 12 | 67 |  |
|  | Prospective | 3 | 17 |  |
|  | Not clear | 1 | 6 |  |
|  | *Cohort design* |  |  |  |
|  | Retrospective | 0 | 0 |  |
|  | Prospective | 1 | 6 |  |
|  | Not clear | 0 | 0 |  |
|  | *Other design* | 1 | 6 |  |
| *Year of Publication* | **Year of publication** |  |  |  |
|  | 2004 | 1 | 6 |  |
|  | 2005 | 1 | 6 |  |
|  | 2006 | 2 | 11 |  |
|  | 2007 | 4 | 22 |  |
|  | 2008 | 0 | 0 |  |
|  | 2009 | 1 | 6 |  |
|  | 2010 | 1 | 6 |  |
|  | 2011 | 4 | 22 |  |
|  | 2012 | 1 | 6 |  |
|  | 2013 | 1 | 6 |  |
|  | 2014 | 2 | 11 |  |
| *Data Collection* | **Consecutive attendances studied** |  |  |  |
|  | Yes | 18 | 100 |  |
|  | No | 0 | 0 |  |
|  | **Duration of data collection** |  |  |  |
|  | < 6 months | 7 | 39 |  |
|  | Between 6 months and 1 year | 6 | 33 |  |
|  | Between 1 year and 3 years | 1 | 6 |  |
|  | Between 3 years and 5 years | 1 | 6 |  |
|  | > 5 years | 3 | 17 |  |
| *Patient Selection* | **Level of data reporting** |  |  |  |
|  | Episodes | 8 | 44 |  |
|  | Patients | 6 | 33 |  |
|  | Both | 4 | 22 |  |
|  | **Target population** |  | 0 |  |
|  | All mental health-related ED attendances | 12 | 67 |  |
|  | Frequent mental health-related attendances | 3 | 17 |  |
|  | ED attendees under section | 3 | 17 |  |
|  | **Instrument used to code mental health conditions** |  |  |  |
|  | ICD - 9/10 | 7 | 39 |  |
|  | DSM – IV | 3 | 17 |  |
|  | Health professional’s assessment | 3 | 17 |  |
|  | Other | 1 | 6 |  |
|  | Unclear | 4 | 22 |  |
|  | **Sample size (Mental health attendances or patients)** |  | 0 |  |
|  | 10-100 | 2 | 11 |  |
|  | 100-500 | 4 | 22 |  |
|  | 500-2500 | 7 | 39 |  |
|  | >2500 | 3 | 17 |  |
|  |  |  |  |  |
