# Supplementary material for: Epidemiology of Mental Health Attendances at Emergency Departments: Systematic Review and Meta-Analysis
Source: PLoS One. 2016 Apr 27;11(4):e0154449. doi: 10.1371/journal.pone.0154449 (PMC4847792; doi:10.1371/journal.pone.0154449)
Supplement: S4 Appendix — (DOCX) [file pone.0154449.s004.docx]

**Appendix 4 Summary of main characteristics of included studies (n=18).**

|  | **Focus of the study** | **Study design** | | **Country** | **No. of sites** | **Urbanisation** | **Data source(s)** | **Consecutive attendances** | **Year(s) of data collection** | **Time span (days)** | **Included cases** | | **Overall quality** |
| --- | --- | --- | --- | --- | --- | --- | --- | --- | --- | --- | --- | --- | --- |
|  |  |  |  |  |  |  |  |  |  |  | **Episodes** | **People** |  |
| Cassar^33^ (2012) | **All MH attenders** | Retrospective | Cross-sectional | England | 1 | Urban | Routine ED database, medical records at the ED, clinicians | Yes | 1997 | 92 | 565 | NR | Poor |
| Fry^20^  (2004) | **All MH attenders** | Retrospective | Cross-sectional | Australia | 1 | Urban | Medical records at the ED | Yes | 2002-2003 | 365 | NR | 1076 | Fair |
| Johansen^35^  (2009) | **All MH attenders** | Retrospective | Cross-sectional | Norway | 2 | Mixed | Medical records at the ED | Yes | 2006 | 365 | 728 | NR | Fair |
| Kalucy^21^  (2005) | **All MH attenders** | Retrospective | Cross-sectional | Australia | 1 | Suburban | Medical records at the ED | Yes | 1994-2003 | 3652 | NR | NR | Poor |
| Knott^22^  (2007) | **All MH attenders** | Retrospective | Cross-sectional | Australia | 5 | Mixed | Medical records at the ED | Yes | 2004 | 153 | 3857 | 3702 | Good |
| Pascual^29^  (2007) | **All MH attenders** | Prospective | Cross-sectional | Spain | 1 (psych) | Urban | Routine ED database | Yes | 2002-2006 | 1461 | 11578 | NR | Good |
| Pereira^18^  (2013) | **All MH attenders** | Retrospective | Cross-sectional | Portugal | 1 (psych) | Urban | Medical records at the ED | Yes | 2010 | 181 | 4537 | NR | Fair |
| Perez-Rodriguez^30^  (2006) | **All MH attenders** | Prospective | Cross-sectional | Spain | 1 (psych) | Urban | Medical records at the ED, clinicians, patients | Yes | 2003 | 265 | 1511 | NR | Poor |
| Prats^19^  (2011) | **All MH attenders (age 65+)** | Not clear | Cross-sectional | Spain | 1 (psych) | Urban | NR | Yes | 2010 | 31 | NR | 36 | Poor |
| Shafiei^23^  (2011) | **All MH attenders** | Retrospective | Cross-sectional | Australia | 1 | Suburban | Routine ED database, medical records at the ED | Yes | Time 1: 2008  Time 2: 2009 | 31  31 | NR | 290 | Fair |
| Tankel^24^  (2011) | **All MH attenders** | Retrospective | Cross-sectional | Australia | 36 | Mixed | Medical records at the ED | Yes | 1999-2006 | 2922 | 290606 | NR | Fair |
|  |  |  |  |  |  |  |  |  |  |  |  |  |  |
| Brunero^25^  (2007) | **Frequent MH attenders** | Retrospective | Cross-sectional | Australia | 1 | Urban | Routine ED database | Yes | 2002-2003 | 365 | 1076 | 869 | Fair |
| Chaput^31^  (2007) | **Frequent attenders (age 20+)** | Prospective | Cohort | Canada | 1 (psych) | Urban | Routine ED database | Yes | 1985-2000 | 5679 | NR | 3853 | Fair |
| Okorie^34^  (2011) | **Frequent attenders** | Retrospective | Case-control | Ireland | 1 | Urban | Routine ED database, medical records at the ED | Yes | 2007 | 184 | 639 | 489 | Fair |
|  |  |  |  |  |  |  |  |  |  |  |  |  |  |
| Al-Khafaji^26^  (2014) | **Attenders under section (age 16+)** | Retrospective | Cross-sectional | Australia | 1 | Suburban | Routine ED database, medical records at the ED | Yes | 2009 | 365 | 197 | 164 | Good |
| Brierley^27^  (2010) | **Attenders under section** | Retrospective | Cross-sectional | Australia | 1 | Other | Routine ED database, medical records at the ED | Yes | 2008 | 183 | 168 | NR | Fair |
| Kang^32^  (2014) | **Attenders under section (age 18+)** | Retrospective | Cross-sectional | Canada | 2 | NR | Medical records at the ED | Yes | 2012 | 366 | 1487 | NR | Fair |
|  |  |  |  |  |  |  |  |  |  |  |  |  |  |
| Lee^28^  (2006) | **Police presentations** | Prospective | Cross-sectional | Australia | 1 | Urban | Clinicians | Yes | 2002-2004 | 731 | 452 | NR | Fair |
