# Supplementary material for: Epidemiology of Mental Health Attendances at Emergency Departments: Systematic Review and Meta-Analysis
Source: PLoS One. 2016 Apr 27;11(4):e0154449. doi: 10.1371/journal.pone.0154449 (PMC4847792; doi:10.1371/journal.pone.0154449)
Supplement: S6 Appendix — (DOCX) [file pone.0154449.s006.docx]

**Appendix S6 Excluded articles with reasons (n=186)**

**Study conducted in an ineligible country (=4)**

Kwok C L; Yip P S. F; Gunnell D, Kuo C J; Chen Y Y. Non-fatal repetition of self-harm in taipei city,Taiwan: Cohort study. *British Journal of Psychiatry* 2014; 204: 376-382.

Song J Y; Choi J S; Lee J Y; Jung H Y; Lee H W; Hong K J; Song K J; Hwang J Y. The characteristics of people who attempted suicide during alcohol intoxication. *Psychotherapy and Psychosomatics*. 2013; 82: 106.

Ukai K, Mizuno Y. Physical complications for elderly inpatients with senile dementia in the Imaise Branch of Ichinomiya City Hospital. *Psychogeriatrics:The Official Journal of the Japanese Psychogeriatric Society*. 2009; 9: 167-72.

Ceilley J W; Douaihy A B; Salloum I M. Prevalence and impact of medical disorders in hospitalized psychiatric patients with comorbid substance use disorders. *Addictive Disorders and their Treatment*. 2005; 4: 65-70.

**Focus was an ineligible clinical condition (n=8)**

Bieler G, Paroz S, Faouzi M, Trueb L, Vaucher P, Althaus F, Daeppen J B; Bodenmann P. Social and medical vulnerability factors of emergency department frequent users in a universal health insurance system. *Academic Emergency Medicine* 2012; 19: 63-68.

Cherpitel C J; Ye Y, Bond J, Rehm J, Poznyak V, Macdonald S, Stafström M, Hao W. Multi-level analysis of alcohol-related injury among emergency department patients: a cross-national study: a research report from the Emergency Room Collaborative Alcohol Analysis Project (ERCAAP) and the WHO Collaborative Study on Alcohol and Injuries. *Addiction* 2005; 100: 1840-1850.

Cherpitel C J; Ye Y, Bond J, Rehm J, Cremonte M, Neves O, Moskalewicz J, Swiatkiewicz G, Giesbrecht N. The effect of alcohol consumption on emergency department services use among injured patients: A cross-national emergency room study. *Journal of Studies on Alcohol* 2006; 67: 890-897.

Cherpitel Cheryl J; Ye Yu, Bond Jason, Borges Guilherme, Chou Patricia, Nilsen Per, Ruan June, Xiang Xiaojun. Multi-level analysis of alcohol-related injury and drinking pattern: emergency department data from 19 countries*^†^. *Addiction* 2012; 107: 1263-1272.

Ciano R, Barbagallo C, Taboga F, Sophia Z, Meduri M, Canalaz F, Ragogna M, Balestrieri M, Rodolfo S, Gangi F. The unexplained chest pain: Preliminary data of an observational study. *European Psychiatry* 2012; 27: doi:10.1016/S0924-9338(12)74414-0

Macdonald S, Cherpitel C J; DeSouza A, Stockwell T, Borges G, Giesbrecht N. Variations of alcohol impairment in different types, causes and contexts of injuries: results of emergency room studies from 16 countries. *Accident Analysis & Prevention* 2006; 38: 1107-12.

Nordqvist C, Holmqvist M, Nilsen P, Bendtsen P, Lindqvist K. Usual drinking patterns and non-fatal injury among patients seeking emergency care. *Public Health* 2006; 120: 1064-73.

OCallaghan B, Ogorman S. Patients who walk out from our Emergency Departments; is alcohol an issue?. *BMC Proceedings* 2012;6:P56

**Study conducted in clinical setting other than ED (n=41)**

Amiel-Lebigre F. Psycho-social determinants for psychiatric hospitalisation for neurotic disorders in women - A case control study. *Social Psychiatry and Psychiatric Epidemiology* 2003; 38: 317-325.

Belgamwar R B; Hodgson R E; Waters K. Trends and characteristics of deliberate self-harm hospital presentations in an English County. *International Journal of Psychiatry in Clinical Practice* 2006; 10: 59-63.

Bjornaas M A; Teige B, Hovda K E; Ekeberg O, Heyerdahl F, Jacobsen D. Fatal poisonings in Oslo: a one-year observational study. *BMC Emergency Medicine* 2010; 10: 13.

Boyd J, Randell T, Luurila H, Kuisma M. Serious overdoses involving buprenorphine in Helsinki. *Acta Anaesthesiologica Scandinavica* 2003; 47: 1031-1033.

Brakoulias V, Mandali R, Seymour J, Sammut P, Starcevic V. Characteristics of admissions to a recently opened Psychiatric Emergency Care Centre. *Australasian Psychiatry* 2010; 18: 326-9.

Brooker C, Ricketts T, Bennett S, Lemme F. Admission decisions following contact with an emergency mental health assessment and intervention service. *Journal of Clinical Nursing* 2007; 16: 1313-1322.

Cailhol L, Moraru R, Barbe R, Moncany A H; Garcin S, Lazignac C, Damsa C. Month of birth, a risk factor for violent behaviour in suicidal patients admitted in emergency? *Psychiatric Quarterly* 2009; 80: 125-130.

Cerejeira J, Firmino H, Boto I, Rita H, Santos G, Teixeira J, Vale L, Abrantes P, Vaz Serra, A. Psychiatric emergency service use in coimbra university hospitals: Results from a 6-month crosssectional study sample. *European Psychiatry* 2009; 24: S899.

Deraas T S; Hansen V, Giaever A, Olstad R (2006) Acute psychiatric admissions from an out-of-hours Casualty Clinic; how do referring doctors and admitting specialists agree? *BMC Health Services Research* 2006; 6: 41.

Dietze P M; Cvetkovski S, Barratt M J; Clemens S. Patterns and incidence of gamma-hydroxybutyrate (GHB)-related ambulance attendances in Melbourne, Victoria. *Medical Journal of Australia* 2008; 188: 709-11.

Doak M W; Nixon A C; Lupton D J; Waring WS. Self-poisoning in older adults: patterns of drug ingestion and clinical outcomes. *Age & Ageing* 2009; 38: 407-11.

Finseth P I; Morken G, Andreassen O A; Malt U F; Vaaler A E. Risk factors of suicidal behaviour in acutely admitted bipolar disorder patients. *Bipolar Disorders* 2011; 13: 45.

Ginos J M; Carcoles D, Malagan A, Arcega J M; Garnier C, Gasque S, Merino A, Bulbena A. Differences between Affective and other disorders in emergencies. *International Clinical Psychopharmacology* 2011; 26: e41.

Hayward M, Moran P. Personality disorder and pathways to inpatient psychiatric care. *Social Psychiatry & Psychiatric Epidemiology* 2007; 42: 502-6.

Heyerdahl F, Bjornas M, Hovda K E; Skog K, Opdahl A, Wium C, Ekeberg O, Jacobsen D. Acute poisonings treated in hospitals in Oslo: A one-year prospective study (II): Clinical outcome. *Clinical Toxicology* 2008; 46: 42-49.

Hovda K E; Bjornaas M A; Skog K, Opdahl A, Drottning P, Ekeberg O, Jacobsen D. Acute poisonings treated in hospitals in Oslo: A one-year prospective study (I): Pattern of poisoning. *Clinical Toxicology* 2008; 46: 35-41.

Johansen I H; Morken T, Hunskaar S. Contacts related to mental illness and substance abuse in primary health care: a cross-sectional study comparing patients' use of daytime versus out-of-hours primary care in Norway. *Scandinavian Journal of Primary Health Care* 2010; 28: 160-165.

Joubert L, Petrakis M, Cementon E. Suicide Attempt Presentations at the Emergency Department: Outcomes From a Pilot Study Examining Precipitating Factors in Deliberate Self-Harm and Issues in Primary Care Physician Management. *Social Work in Health Care* 2012; 51: 66-76.

Ledoux Y, Minner P. Occasional and frequent repeaters in a psychiatric emergency room. *Social Psychiatry & Psychiatric Epidemiology* 2006; 41: 115-21.

Lejoyeux M, Boulenguiez S, Fichelle A, McLoughlin M, Claudon M, Ades J. Alcohol dependence among patients admitted to psychiatric emergency services. *General Hospital Psychiatry* 2000; 22: 206-212.

Lejoyeux M, Huet F, Claudon M, Fichelle A, Casalino E, Lequen V. Characteristics of suicide attempts preceded by alcohol consumption. *Archives of Suicide Research* 2008; 12: 30-38.

Lejoyeux M, Gastal D, Bergeret A, Casalino E, Lequen V, Guillermet S. Alcohol use disorders among patients examined in emergency departments after a suicide attempt. *European Addiction Research* 2011; 18: 26-33.

Mosel K A; Gerace A, Muir-Cochrane E. Retrospective analysis of absconding behaviour by acute care consumers in one psychiatric hospital campus in Australia. *International Journal of Mental Health Nursing* 2010; 19: 177-185.

Nrugham L, Mehlum L. Differences and similarities of number of life events among patients admitted after attempted suicide. *European Psychiatry* 2010; 25(S1): 1365.

O'Farrell A, Allwright S, Downey J, Bedford D, Howell F. The burden of alcohol misuse on emergency in-patient hospital admissions among residents from a health board region in Ireland. *Addiction* 2004; 99: 1279-85.

O'Loughlin S, Sherwood J. A 20-year review of trends in deliberate self-harm in a British town, 1981-2000. *Social Psychiatry and Psychiatric Epidemiology* 2005;40: 446-453.

O'Neill A, Casey P, Minton R. The homeless mentally ill - An audit from an inner city hospital. *Irish Journal of Psychological Medicine* 2007; 24: 62-66.

Sands N, Elsom S, Gerdtz M, Henderson K, Keppich-Arnold S, Droste N, Prematunga R K; Wereta Z W. Identifying the core competencies of mental health telephone triage. *Journal of Clinical Nursing* 2013; 22: 3203-3216.

Spurrell M, Hatfield B, Perry A. Characteristics of patients presenting for emergency psychiatric assessment at an English hospital. *Psychiatric Services* 2003; 54: 240-5.

Steel R M; McKay I M. Pathways to psychiatric admission: a study of 100 consecutive admissions to south Glasgow acute adult psychiatric wards. *Health Bulletin* 2000; 58: 112-117.

Suominen K, Isometsa E, Martunnen M, Ostamo A, Lonnqvist J. Health care contacts before and after attempted suicide among adolescent and young adult versus older suicide attempters. *Psychological Medicine* 2004; 34: 313-21.

Suominen K H; Isometsa E T; Lonnqvist J K. Attempted suicide and psychiatric consultation. *European Psychiatry* 2004; 19: 140-145.

Tauch D, Winkel S, Quante A. Psychiatric consultations and therapy recommendations following a suicide attempt in a general hospital and their associations with selected parameters in a 1-year period. *International Journal of Psychiatry in Clinical Practice* 2014; 18: 118-124.

Ul Haq, S , Ratnayake T, Agius M. Mental health assessment unit audit. *Psychiatria Danubina* 2010; 22: S23-S25.

Villars H, Dupuy C, Soler P, Gardette V, Soto ME; Gillette S, Nourhashemi F, Vellas B. A follow-up intervention in severely demented patients after discharge from a special Alzheimer acute care unit: Impact on early emergency room re-hospitalization rate. *International Journal of Geriatric Psychiatry* 2013; 28: 1131-1140.

Wichmann S, Nielsen SL; Siersma VD; Rasmussen LS. Risk factors for 48-hours mortality after prehospital treatment of opioid overdose. *Emergency Medicine Journal* 2013; 30: 223-5.

Wiktorsson S, Runeson B, Skoog I, Ostling S, Waern M. Attempted suicide in the elderly: characteristics of suicide attempters 70 years and older and a general population comparison group. *American Journal of Geriatric Psychiatry* 2010; 18: 57-67.

Williamson T, Flowers J, Cooke M. Quantifying emergency department admission rates for people with a learning disability. *Emergency Medicine Journal*. 2012; 29: 771-2.

Wood DM; Button J, Ashraf T, Walker S, Greene SL; Drake N, Ramsey J, Holt DW; Dargan PI. What evidence is there that the UK should tackle the potential emerging threat of methamphetamine toxicity rather than established recreational drugs such as MDMA ('ecstasy')? *QJM*  2008; 101(3): 207-13

Zanone Poma, S , Toniolo E, Grossi A, Pizzo R, Cocchio S, Baldo V. Epidemiology of suicide attempts in a psychiatric setting in Northern Italy. *Journal of Psychopathology* 2013; 19: 119-125.

**Not an epidemiological study of ED attendances (n=33)**

Andrew E, Irestedt B, Hurri T, Jacobsen P, Gudjonsdottir G. Mortality and morbidity of poisonings in the Nordic countries in 2002. *Clinical Toxicology* 2008; 46: 310-313.

Carroll R, Metcalfe C, Gunnell D. Hospital presenting self-harm and risk of fatal and non- fatal repetition: Systematic review and meta-analysis. *PLoS ONE* 2014; DOI: 10.1371/journal.pone.0089944

Cebria AI; Parra I, Pamias M, Escayola A, Garcia-Pares G, Punti J, Laredo A, Valles V, Cavero M, Oliva J C; Hegerl U, Perez-Sola V, Palao DJ. Effectiveness of a telephone management programme for patients discharged from an emergency department after a suicide attempt: Controlled study in a Spanish population. *Journal of Affective Disorders* 2013; 147: 269-276.

Chambers C, Chiu S, Katic M, Kiss A, Redelmeier D A; Levinson W, Hwang SW. High utilizers of emergency health services in a population-based cohort of homeless adults. *American Journal of Public Health* 2013; 103 Suppl 2: S302-310.

Cooper J, Kapur N, Webb R, Lawlor M, Guthrie E, Mackway-Jones K, Appleby L. Suicide after deliberate self-harm: A 4-year cohort study. *American Journal of Psychiatry* 2005; 162: 297-303.

Cooper J, Murphy E, Bergen H, Casey D, Hawton K, Owens D, Lilley R, Noble R, Kapur N. The effect of using NHS number as the unique identifier for patients who self-harm: A multi-centre descriptive study. *Clinical Practice and Epidemiology in Mental Health* 2007; 3: 16.

Czernin S, Vogel M, Bourgnon J C; Muheim F, Reichelt M, Stoppe G. Direct costs of suicide attempts in Basel, Switzerland. *European Psychiatry* 2010; 25(S1): 1685.

Da Cruz , D , Pearson A, Saini P, Miles C, While D, Swinson N, Williams A, Shaw J, Appleby L, Kapur N. Emergency department contact prior to suicide in mental health patients. *Emergency Medicine Journal* 2011; 28: 467-71.

Downes M A; Healy P, Page C B; Bryant J L; Isbister G K. Structured team approach to the agitated patient in the emergency department. *Emergency Medicine Australasia* 2009; 21: 196-202.

Greene S L; Wood D M; Gawarammana I B; Warren-Gash C, Drake N, Jones A L; Dargan P I. Improvement in the management of acutely poisoned patients using an electronic database, prospective audit and targeted educational intervention. *Postgraduate Medical Journal* 2008; 84: 603-608.

Gunnell D, Bennewith O, Hawton K, Simkin S, Kapur N. The epidemiology and prevention of suicide by hanging: A systematic review. *International Journal of Epidemiology* 2005; 34: 433-442.

Hughes N R; Houghton N, Nadeem H, Bell J, McDonald S, Glynn N, Scarfe C, MacKay B, Rogers A, Walters M, Smith M, McDonald A, Dalton D. Salford alcohol assertive outreach team: A new model for reducing alcohol-related admissions. *Frontline Gastroenterology* 2013; 4: 130-134.

Husain M I; Waheed W, Husain N. Self-harm in British South Asian women: Psychosocial correlates and strategies for prevention. *Annals of General Psychiatry* 2006; 5: 7.

Kaltiala-Heino R, Tuohimaki C, Korkeila J, Lehtinen V. Reasons for using seclusion and restraint in psychiatric inpatient care. *International Journal of Law and Psychiatry* 2003; 26: 139-149.

Kinmond K S; Bent M. Attendance for self-harm in a West Midlands hospital A&E department. *British Journal of Nursing* 2000; 9(4): 215-220.

Kwon C, Liu M, Quan H, Thoo V, Wiebe S, Jette N. Motor vehicle accidents, suicides, and assaults in epilepsy: A population-based study. *Neurology* 2011; 76: 801-806.

Lloyd B, Matthews S, Livingston M, Jayasekara H, Smith K. Alcohol intoxication in the context of major public holidays, sporting and social events: a time-series analysis in Melbourne, Australia, 2000-2009. *Addiction* 2013; 108: 701-709.

Mulder C, De Leeuw, R. The organisation of emergency psychiatry in the Netherlands. *European Psychiatry* 2011; 26(S1): 2002

Nordentoft M, Sogaard M. Registration, psychiatric evaluation and adherence to psychiatric treatment after suicide attempt. *Nordic Journal of Psychiatry* 2005; 59: 213-216.

O'Daly K, Davies S. Where's the psychiatry in the emergency department? *Australian and New Zealand Journal of Psychiatry* 2012; 46: 61.

Parra Uribe, I , Blasco-Fontecilla H, Garcia-Pares G, Giro Batalla, M , Llorens Capdevila, M , Cebria Meca, A , de Leon-Martinez , V , Perez-Sola V, Palao Vidal, D J. Attempted and completed suicide: not what we expected?. *Journal of Affective Disorders* 2013; 150: 840-6.

Perez-Rodriguez M M; Baca-Garcia E, Quintero-Gutierrez F J; Gonzalez G, Saiz-Gonzalez D, Botillo C, Basurte-Villamor I, Sevilla J, Gonzalez De Rivera; J L. Demand for psychiatric emergency services and immigration. Findings in a Spanish hospital during the year 2003. *European Journal of Public Health* 2006; 16: 383-387.

Riedi G, Mathur A, Seguin M, Bousquet B, Czapla P, Charpentier S, Genestal M, Cailhol L, Birmes P. Alcohol and repeated deliberate self-harm: Preliminary Results of the French Cohort Study of Risk for Repeated Incomplete Suicides. *Crisis* 2012; 33: 358-363.

Sakinofsky I. Attendance at accident and emergency for deliberate self harm predicts increased risk of suicide, especially in women. *Evidence Based Mental Health* 2005; 8: 97-97.

Sebbane M, Claret P G; Jreige R, Dumont R, Lefebvre S, Rubenovitch J, Mercier G, Eledjam J J; de la Coussaye , JE. Breath analyzer screening of emergency department patients suspected of alcohol intoxication. *Journal of Emergency Medicine* 2012; 43: 747-53.

Shajahan P, Agnew T. Availability of patient records and psychiatric admission rate. *Psychiatric Bulletin* 2005; 30: 449-451.

Skogman K, Alsen M, Ojehagen A. Sex differences in risk factors for suicide after attempted suicide-a follow-up study of 1052 suicide attempters. *Social Psychiatry & Psychiatric Epidemiology* 2004; 39: 113-20.

Smeijers L, Nyklicek I, Notten P J; Pedersen S S; Van De Pas , H , Kop W J. Independent association of anxiety with emergency room admission for non-cardiac chest pain. *Psychosomatic Medicine* 2012; 74 (3): A58.

Spittal M J; Pirkis J, Miller M, Carter G, Studdert D M. The Repeated Episodes of Self-Harm (RESH) score: A tool for predicting risk of future episodes of self-harm by hospital patients. *Journal of Affective Disorders* 2014; 161: 36-42.

Suominen K, Isometsa E, Haukka J, Lonnqvist J. Substance use and male gender as risk factors for deaths and suicide - A 5-year follow-up study after deliberate self-harm. *Social Psychiatry and Psychiatric Epidemiology* 2004; 39: 720-724.

Vandyk Amanda Digel; Harrison Margaret B; Van DenKerkhof , Elizabeth G, Graham Ian D; Ross-White Amanda. Frequent emergency department use by individuals seeking mental healthcare: A systematic search and review. *Archives of Psychiatric Nursing* 2013; 27: 171-178.

Wood DM; Greene SL; Dargan PI. Five-year trends in self-reported recreational drugs associated with presentation to a UK emergency department with suspected drug-related toxicity. *European Journal of Emergency Medicine* 2013; 20: 263-7.

Wood D M; Dargan P I. Toxicosurveillance of Novel Psychoactive Substances: An emergency department perspective and the role of the European Drug Emergencies Network (Euro-DEN) project. *Clinical Toxicology* 2014; 52: 350.

**Study of an ineligible population group (n=8)**

Bhugra D, Thompson N, Singh J, Fellow-Smith E. Inception rates of deliberate self-harm among adolescents in West London. *International Journal of Social Psychiatry* 2003; 49: 247-250.

Dent A, Hunter G, Webster AP. The impact of frequent attenders on a UK emergency department. *European Journal of Emergency Medicine* 2010; 17: 332-336.

Henson VL; Vickery DS. Patient self-discharge from the emergency department: Who is at risk? *Emergency Medicine Journal* 2005; 22: 499-501.

Khav N, Weiland T, Jelinek G, Salzberg M, Knott J. Depression symptoms and risk factors in adult emergency department patients: A multi-site cross-sectional prevalence survey. *Emergency Medicine Australasia* 2012; 24: 15.

Kinner S A; Alati R, Watt K, Najman J M; Fowler G, Green D. Substance misuse, anxiety and depression and urgency of presentation to a public emergency department in Australia. *Emergency Medicine Australasia* 2005; 17: 363-370.

Mola Gibert, M , Pou Calvo, R , Montesinos Rueda, L , Gine Serven, E , Alvarez Gomez, A , Batlle De Santiago, E. Detection of substances of abuse in emergency department: Is there an increasing the use of benzodiazepines? *European Neuropsychopharmacology* 2010; 20: S325-S326.

Nirui M, Dudley M, Adily A, Ferson M. Identification of youth who have deliberately self poisoned--a medical records audit in university teaching hospitals. *Archives of Suicide Research* 2002; 6: 143-153.

Wood DM; Greene SL; Dargan PI. A study assessing representation in patients presenting to the emergency department with acute recreational drug toxicity. *Clinical Toxicology* 2010; 48 (3): 305-306.

**Focus was alcohol or illicit drug use (n=29)**

Allely P, Graham W, McDonnell M, Spedding R. Alcohol levels in the emergency department: A worrying trend. *Emergency Medicine Journal* 2006; 23: 707-708.

Assad-Sangabi A, Smith R, Cleminson J, Jones L, Elphick D. Brief intervention reduces future hospital attendance in hazardous alcohol drinkers. *Gut* 2012; 61: A74.

Baune BT; Mikolajczyk RT; Reymann G, Duesterhaus A, Fleck S, Kratz H, Sundermann U. A 6-months assessment of the alcohol-related clinical burden at emergency rooms (ERs) in 11 acute care hospitals of an urban area in Germany. *BMC Health Services Research* 2005; 5: 73.

Benger J, Carter R. Could inter-agency working reduce emergency department attendances due to alcohol consumption? *Emergency Medicine Journal* 2008; 25: 331-334.

Binks S, Hoskins R, Salmon D, Benger J. Prevalence and healthcare burden of illegal drug use among emergency department patients. *Emergency Medicine Journal* 2005; 22: 872-873.

Bodmer M, Bruggisser M, Enzler F, Liechti M. Demographics, co-use of illicit drugs, and clinical presentation of patients with acute cocaine intoxication presenting to a swiss emergency department. *Clinical Toxicology* 2012; 50 (4): 343-344.

Brugal M T, Domingo-Salvany A, Diaz De Quijano E, Torralba L. Prevalence of problematic cocaine consumption in a city of southern Europe, using capture-recapture with a single list. *Journal of Urban Health* 2004; 81: 416-427.

Carballo JJ, Oquendo M A, Garcia-Moreno M, Poza B, Giner L, Baca E, Zalsman G, Roche A M, Sher L. Demographic and clinical features of adolescents and young adults with alcohol-related disorders admitted to the Psychiatric Emergency Room. *International Journal of Adolescent Medicine and Health* 2006; 18: 87-96.

Degenhardt L J; Conroy E, Gilmour S, Hall W D. The effect of a reduction in heroin supply on fatal and non-fatal drug overdoses in New South Wales, Australia. *Medical Journal of Australia* 2005; 182: 20-3.

Everitt R, Jones P. Changing the minimum legal drinking age--its effect on a central city emergency department. *New Zealand Medical Journal* 2002; 115: 9-11.

Fatovich DM; Bartu A, Daly F. A prospective study of non-fatal heroin overdose. *Journal of Substance Use* 2008; 13: 299-307.

Galicia M, Nogue S, Miro O. Liquid ecstasy intoxication: clinical features of 505 consecutive emergency department patients. *Emergency Medicine Journal* 2011; 28: 462-6.

Gibbs T, Ross L. Illicit drug use related attendances at accident and emergency services in Aberdeen: a prospective six month survey. *Health Bulletin* 2000; 58: 170-176.

Gudjonsdottir G A; Thordardottir A M; Kristinsson J. Acute poisonings in Iceland: A prospective study of poisonings presenting to the emergency department at Landspitali University Hospital. *Clinical Toxicology* 2014; 52: 312.

Haberkern M, Exadaktylos AK, Marty H. Alcohol intoxication at a university hospital acute medicine unit - with special consideration of young adults: An 8-year observational study from Switzerland. *Emergency Medicine Journal* 2010; 27: 199-202.

Havard A, Shakeshaft AP, Conigrave KM; Sanson-Fisher RW. The prevalence and characteristics of alcohol-related presentations to emergency departments in rural Australia. *Emergency Medicine Journal* 2011; 28: 290-295.

Horyniak D, Degenhardt L, Smit D V; Munir V, Johnston J, Fry C, Dietze P. Pattern and characteristics of ecstasy and related drug (ERD) presentations at two hospital emergency departments, Melbourne, Australia, 2008-2010. *Emergency Medicine Journal* 2014; 31: 317-322.

Kristinsson J, Palsson R, Gudjonsdottir G A; Blondal M, Gudmundsson S, Snook CP. Acute poisonings in Iceland: A prospective nationwide study. *Clinical Toxicology* 2008; 46: 126-132.

Lee V, Fergus Kerr, J , Braitberg G, Louis W J; O'Callaghan C J; Frauman A G; Mashford M L. Impact of a toxicology service on a metropolitan teaching hospital. *Emergency Medicine* 2001; 13: 37-42.

Newton A, Sarker SJ, Pahal GS; van den Bergh E, Young C. Impact of the new UK licensing law on emergency hospital attendances: a cohort study. *Emergency Medicine Journal* 2007; 24: 532-4.

Pain S, Chavant F, Fauconneau B, Garnier E, Lardeur J Y; Perault-Pochat M C. Intoxication with opioid analgesic drugs: Descriptive study in a Hospital Emergency department. *Fundamental and Clinical Pharmacology* 2014; 28: 48.

Peraro L, Lobello S, Rosa-Rizzotto E, Caroli D, Polato F, De Lazzari F. Binge drinking, alcohol abuse and dependence: Screening study in an emergency department. *Alcohol and Alcoholism* 2013; 48: i57.

Pirmohamed M, Brown C, Owens L, Luke C, Gilmore I T; Breckenridge A M; Park B K. The burden of alcohol misuse on an inner-city general hospital. *QJM - Monthly Journal of the Association of Physicians* 2000; 93: 291-295.

Tjipto AC; Taylor D; Liew H. Alcohol use among young adults presenting to the emergency department. *Emergency Medicine Australasia* 2006;18: 125-130.

Verelst S, Moonen P, Desruelles D, Gillet J. Emergency department visits due to alcohol intoxication: Characteristics of patients and impact on the emergency room. *Alcohol and Alcoholism* 2012; 47: 433-438.

Vermes A, Roelofsen EE, Sabadi G, van den Berg B, de Quelerij M, Vulto AG. Intoxication with therapeutic and illicit drug substances and hospital admission to a Dutch university hospital. *Netherlands Journal of Medicine* 2003; 61: 168-72.

Zannoni M, Ricci G, Codogni R, Tobaldini C, Puglisi S, Formaglio E, Serafini V, Cigolini D. Elderly acute intoxications in the emergency department from 2005 to 2011: Patterns and outcomes. *Clinical Toxicology* 2012; 50 (4): 331.

**Focus was a specific mental health condition (n=3)**

Psychosis: Florea R, Ho Quoc, H , Courtet P H; (2010) Reasons for admission of psychotic patients to psychiatric emergency services. *European Neuropsychopharmacology*. 20: S451-S452.

Psychosis: Latt N, Jurd S, Tennant C, Lewis J, MacKen L, Joseph A, Grochulski A, Long L (2011) Alcohol and substance use by patients with psychosis presenting to an emergency department: Changing patterns. *Australasian Psychiatry*. 19: 354-359.

OCD: Mota Rodriguez, M J, Pampin Alfonso, A , Portillo Diez, J , De Usabel Guzman, P , Perez Garcia, M (2011) Patients profile with obsessive-compulsive disorder attending to psychiatric emergency department. *European Psychiatry*. 26: .

**Focus was self-harm, attempted suicide or overdose (n=57)**

Ardagh M, Flood D, Tait C. Limiting the use of gastrointestinal decontamination does not worsen the outcome from deliberate self-poisoning. *New Zealand Medical Journal* 2001; 114: 423-425.

Armstrong TM; Davies MS; Kitching G, Waring WS. Comparative drug dose and drug combinations in patients that present to hospital due to self-poisoning. *Basic & Clinical Pharmacology & Toxicology* 2012; 111: 356-60.

Artieda Urrutia P, Gomez-Arnau Ramirez J, Sanchez Paez P, Gomez Soler R , De La Vega Sanchez D. Personality disorder as a relevant factor to the management of drug overdoses in the emergency service. *European Psychiatry* 2011; 26(S1): 1606

Barr W, Leitner M, Thomas J. Short shrift for the sane? The hospital management of self-harm patients with and without mental illness. *Journal of Psychiatric & Mental Health Nursing* 2004; 11: 401-6.

Barr W, Leitner M, Thomas J. Self-harm patients who take early discharge from the accident and emergency department: how do they differ from those who stay?. *Accident & Emergency Nursing* 2004; 12: 108-13.

Bastos H, Polido F, Pereira S, Garrido P, Craveiro A. Suicidal behavior in psychiatric emergency room patients. *European Psychiatry* 2012; 27(S1): 1.

Bennett S, Coggan C, Hooper R, Lovell C, Adams P. Presentations by youth to Auckland emergency departments following a suicide attempt. *International Journal of Mental Health Nursing* 2002; 11: 144-153.

Bergen H, Hawton K, Waters K, Cooper J, Kapur N. Epidemiology and trends in non-fatal self-harm in three centres in England: 2000-2007. *British Journal of Psychiatry* 2010; 197: 493-498.

Bilen K, Ottosson C, Castren M, Ponzer S, Ursing C, Ranta P, Ekdahl K, Pettersson H (2011) Deliberate self-harm patients in the emergency department: Factors associated with repeated self-harm among 1524 patients. *Emergency Medicine Journal* 2011; 28: 1019-1025.

Bolger S, O'Connor P, Malone K, Fitzpatrick C. Adolescents with suicidal behaviour: Attendance at A&E and six month follow-up. *Irish Journal of Psychological Medicine* 2004; 21: 78-84.

Bruyndonckx RB, Meulemans AI, Sabbe MB, Kumar AA, Delooz HH. Fatal intentional poisoning cases admitted to the University Hospitals of Leuven, Belgium from 1993 to 1996. *European Journal of Emergency Medicine* 2002; 9: 238-243.

Burillo-Putze G, Munne P, Duenas A, Pinillos M A; Naveiro J M; Cobo J, Alonso J. National multicentre study of acute intoxication in emergency departments of Spain. *European Journal of Emergency Medicine* 2003; 10: 101-104.

Buykx P, Dietze P, Ritter A, Loxley W. Characteristics of medication overdose presentations to the ED: how do they differ from illicit drug overdose and self-harm cases? *Emergency Medicine Journal* 2010; 27: 499-503.

Colman I, Yiannakoulias N, Schopflocher D, Svenson LW, Rosychuk RJ, Rowe BH. Population-based study of medically treated self-inflicted injuries. *Canadian Journal of Emergency Medicine* 2004; 6: 313-320.

Cook R, Allcock R, Johnston M. Self-poisoning: Current trends and practice in a UK teaching hospital. *Clinical Medicine* 2008; 8: 37-40.

Cooper J, Husain N, Webb R, Waheed W, Kapur N, Guthrie E, Appleby L. Self-harm in the UK: Differences between South Asians and Whites in rates, characteristics, provision of service and repetition. *Social Psychiatry and Psychiatric Epidemiology* 2006; 41: 782-788.

Corcoran P, Arensman E, Perry IJ. The area-level association between hospital-treated deliberate self-harm, deprivation and social fragmentation in Ireland. *Journal of Epidemiology and Community Health* 2007; 61: 1050-1055.

Corcoran P, Reulbach U, Keeley HS; Perry IJ; Hawton K, Arensman E. Use of analgesics in intentional drug overdose presentations to hospital before and after the withdrawal of distalgesic from the Irish market. *BMC Clinical Pharmacology* 2010; 10: 6.

Corcoran P, Reulbach U, Perry IJ, Arensman E. Suicide and deliberate self-harm in older Irish adults. *International Psychogeriatrics*. 2010; 22: 1327-1336.

De Munck S, Portzky G, Van Heeringen K. Epidemiological trends in attempted suicide in adolescents and young adults between 1996 and 2004. *Crisis* 2009; 30: 115-119.

Donze N, Fornerod L, Gaspoz A, Bonvin R, Riand-Voide R, Beloeil N, Augsburger M. Toxicology in emergency room in the Valais hospital. *Clinical Chemistry and Laboratory Medicine* 2011; 49 (10): A22.

Donze N, Fornerod L, Chiolero A, Bonvin R, Rossier M F; Augbsurger M. Toxicology in emergency room in the Valais Hospital Between 2007 and 2011. *Clinical Chemistry and Laboratory Medicine* 2012; 50 (5): A164-165.

Elamin M; Peart LC; Hill SL; Thomas SH. Impact of changes in UK management advice for paracetamol overdose on the numbers of adult patients admitted and treated in Newcastle upon Tyne? *Clinical Toxicology* 2014; 52: 305-306.

Elisei S, Verdolini N, Anastasi S. Suicidal attempts among emergency department patients: One-year of clinical experience. *Psychiatria Danubina* 2012; 24: 140-142.

Exiara T, Mavrakanas T A; Papazogiou L, Papazoglou D, Christakidis D, Maltezos E. A prospective study of acute poisonings in a sample of Greek patients. *Central European Journal of Public Health* 2009; 17: 158-160.

Feeney L, Ryan J, Moran P. Parasuicide assessment in the emergency department. *Irish Medical Journal* 2005; 98: 111-113.

Finkelstein Y, Hollands S, Sivilotti ML, Hutson J, Mamdani MM; Juurlink DN. Repetition of deliberate self-poisoning: A population-based cohort study. *Clinical Toxicology* 2013; 51 (4): 334-335.

Graudins A. Paracetamol poisoning in melbourne, Australia: Are we maintaining the NAC of treating paracetamol poisoning? *Clinical Toxicology* 2014; 52: 312-313.

Gunnell D, Bennewith O, Peters TJ, House A, Hawton K. The epidemiology and management of self-harm amongst adults in England. *Journal of Public Health* 2005; 27: 67-73.

Hatcher S, Sharon C, Collins N. Epidemiology of intentional self-harm presenting to four district health boards in New Zealand over 12 months, and comparison with official data. *Australian & New Zealand Journal of Psychiatry* 2009; 43: 659-65.

Hawton K, Bergen H, Casey D, Simkin S, Palmer B, Cooper J, Kapur N, Horrocks J, House A, Lilley R, Noble R, Owens D. Self-harm in England: a tale of three cities. Multicentre study of self-harm. *Social Psychiatry & Psychiatric Epidemiology* 2007; 42: 513-21.

Hawton K, Bergen H, Casey D, Simkin S. General hospital presentations of non-fatal hanging over a 28-year period: case-control study. *British Journal of Psychiatry* 2008; 193: 503-4.

Hawton K, Bergen H, Waters K, Murphy E, Cooper J, Kapur N. Impact of withdrawal of the analgesic co-proxamol on nonfatal self-poisoning in the UK. *Crisis* 2011; 32: 81-87.

Hendrix L, Verelst S, Desruelles D, Gillet J B. Deliberate self-poisoning: Characteristics of patients and impact on the emergency department of a large university hospital. *Emergency Medicine Journal* 2013; 30: e9.

Hepp U, Moergeli H, Trier S N, Milos G, Schnyder U. Attempted suicide: Factors leading to hospitalization. *Canadian Journal of Psychiatry* 2004; 49: 736-742.

Hickey L, Hawton K, Fagg J, Weitzel H. Deliberate self-harm patients who leave the accident and emergency department without a psychiatric assessment: A neglected population at risk of suicide. *Journal of Psychosomatic Research* 2001; 50: 87-93.

Horrocks J, Price S, House A, Owens D. Self-injury attendances in the accident and emergency department: Clinical database study. *British Journal of Psychiatry* 2003; 183: 34-39.

Howson M A; Yates K M; Hatcher S (2008) Re-presentation and suicide rates in emergency department patients who self-harm. *Emergency Medicine Australasia* 2008; 20: 322-7.

Kapur N, Cooper J, Hiroeh U, May C, Appleby L, House A. Emergency department management and outcome for self-poisoning: A cohort study. *General Hospital Psychiatry* 2004; 26: 36-41.

Kapur N, Murphy E, Cooper J, Bergen H, Hawton K, Simkin S, Casey D, Horrocks J, Lilley R, Noble R, Owens D. Psychosocial assessment following self-harm: results from the multi-centre monitoring of self-harm project. *Journal of Affective Disorders* 2008; 106: 285-93.

Karasouli E, Owens D, Abbott RL, Hurst KM, Dennis M. All-cause mortality after non-fatal self-poisoning: a cohort study. *Social Psychiatry and Psychiatric Epidemiology* 2011; 46: 455-462.

Kuehl S, Nelson K, Collings S. Back so soon: rapid re-presentations to the emergency department following intentional self-harm. *The New Zealand Medical Journal* 2012; 125: 70-79.

Larkin C, Corcoran P, Perry I, Arensman E. Severity of hospital-treated self-cutting and risk of future self-harm: A national registry study. *Journal of Mental Health* 2013; 23: 115-119.

Lilley R, Owens D, Horrocks J, House A, Noble R, Bergen H, Hawton K, Casey D, Simkin S, Murphy E, Cooper J, Kapur N. Hospital care and repetition following self-harm: multicentre comparison of self-poisoning and self-injury. *British Journal of Psychiatry* 2008; 192: 440-5.

Lopez JL, Al-Halabi S, Diaz-Mesa EM, Bascaran MT, Bousono M, Garcia-Portilla MP, Saiz PA, Bobes J.Sociodemographic profile and clinical data of suicide attempters from the emergency room of the central hospital of asturias (Northem Spain). *European Psychiatry* 2009; 24: S777.

Lopez-Castroman J, Perez-Rodriguez M, Alegria AA, Artes-Rodriguez A, Freed P, Jollant F, Leiva-Murillo J M, Oquendo MA, de Prado-Cumplido M , Saiz-Ruiz J, Perroud N, Saiz PA, Baca-Garcia E, LopezCastroman J, Blasco-Fontecilla H, Sarchiapone M, Carli V, Courtet P, Jaussent I, Guillaume S, Malafosse A. Distinguishing the relevant features of frequent suicide attempters. *Journal of Psychiatric Research* 2011; 45: 619-625.

Miret M, Morant C, Nuevo R, Jimenez M, Reneses M, Lopez M, Avila C, Ayuso-Mateos JL. Incidence of suicide attempts in Madrid: Characteristics of the attempts and response of the health system. *European Psychiatry* 2009; 24: S804.

Mullins D, MacHale S, Cotter D. Meeting standards set for non self-harm presentations to emergency departments. *Irish Journal of Psychological Medicine* 2011; 28: 185-190.

Myers RP, Li B, Shaheen A. Emergency department visits for acetaminophen overdose: A Canadian population-based epidemiology study (1997-2002). *Canadian Journal of Emergency Medicine* 2007; 9: 267-274.

Perez Garcia M, Portela Traba B, Mozos Ansorena A, Cornes Iglesias JM, Paramo Fernandez M. Assessment of suicide attempts in an emergency service of a general hospital. *European Psychiatry* 2011; 26 (S1): 1635.

Perry IJ, Corcoran P, Fitzgerald AP, Keeley HS, Reulbach U, Arensman E. The incidence and repetition of hospital-treated deliberate self-harm in Ireland. *Journal of Epidemiology & Community Health* 2010; 64: A10-A10.

Prescott K, Stratton R, Freyer A, Hall I, Le Jeune I. Detailed analyses of self-poisoning episodes presenting to a large regional teaching hospital in the UK. *British Journal of Clinical Pharmacology* 2009; 68: 260-268.

Staikowsky F, Theil F, Mercadier P, Candella S, Benais JP. (2004) Change in profile of acute self drug-poisonings over a 10-year period. *Human and Experimental Toxicology* 2004; 23: 507-511.

Suominen K, Lonnqvist J. Determinants of psychiatric hospitalization after attempted suicide. *General Hospital Psychiatry* 2006; 28: 424-30.

Tiatia J, Coggan C. Young Pacifican suicide attempts: a review of emergency department medical records, Auckland, New Zealand. *Pacific Health Dialog* 2001; 8: 124-128.

Tournier M, Grolleau A, Cougnard A, Moore N, Verdoux H, Molimard M. Psychotropic drugs involved in serious intentional drug overdose. *European Psychiatry* 2009; 24: S816.

Wazaify M, Kennedy S, Hughes CM, McElnay JC. Prevalence of over-the-counter drug-related overdoses at Accident and Emergency departments in Northern Ireland - A retrospective evaluation. *Journal of Clinical Pharmacy and Therapeutics* 2005; 30: 39-44.

Wong A, Taylor D M; Ashby K, Robinson J. Changing epidemiology of intentional antidepressant drug overdose in Victoria, Australia. *Australian and New Zealand Journal of Psychiatry* 2010; 44: 759-764.

Yip PS, Hawton K, Liu K, Liu KS, Ng PW, Kam PM, Law YW, Wong T. A study of deliberate self-harm and its repetition among patients presenting to an emergency department. *Crisis* 2011; 32: 217-24.
